# Supplementary material for: Unexpected Decarbonylation of Acylethynylpyrroles under the Action of Cyanomethyl Carbanion: A Robust Access to Ethynylpyrroles
Source: Molecules. 2023 Feb 1;28(3):1389. doi: 10.3390/molecules28031389 (PMC9919934; doi:10.3390/molecules28031389)
Supplement: Supplementary file 1 [file molecules-28-01389-s001.zip › molecules-2126474-supplementary.pdf]

Supplementary Materials

Unexpected Decarbonylation of Acylethynylpyrroles under the Action of Cyanomethyl Carbanion: A Robust Access to Ethynylpyrroles

Denis N. Tomilin [1], Lyubov N. Sobenina [1], Alexandra M. Belogolova [1,2], Alexander B. Trofimov [1,3], Igor A. Ushakov [1] and Boris A. Trofimov [1,*]

[1] A.E. Favorsky Irkutsk Institute of Chemistry, Siberian Branch, Russian Academy of Science, 664033 Irkutsk, Russia

[2] Faculty of Physics, Irkutsk State University, 664003 Irkutsk, Russia

[3] Laboratory of Quantum Chemical Modeling of Molecular Systems, Irkutsk State University, 664003 Irkutsk, Russia

* Correspondence: boris_trofimov@irioch.irk.ru

Contents

General Information S3
Synthesis of ethynylpyrroles **4a**–**k**, ethynylindole **6**, ethynylfuran **8**. General procedure S3
Synthesis of propargyl alcohols **3a,c,d,f** S7
Quantum chemical calculations S9
NMR Spectra of synthesized compounds S13
References S45

General Information

IR spectra were obtained on a "Bruker IFS-25" spectrometer (Bruker, Billerica, MA, USA) (KBr pellets or films in 400-4000 cm^{-1} region). ^1H (400.13 MHz) and ^{13}C (100.6 MHz) NMR spectra were recorded on a "Bruker Avance 400" instrument (Bruker, Billerica, MA, USA) in $CDCl_3$. The assignment of signals in the ^1H NMR spectra was made using COSY and NOESY experiments. Resonance signals of carbon atoms were assigned based on ^1H-^{13}C HSQC and ^1H-^{13}C HMBC experiments. The ^1H chemical shifts (δ) were referenced to the residual solvent protons (7.26 ppm, $CDCl_3$), and the ^{13}C chemical shifts were expressed with respect to the deuterated solvent (77.16 ppm). Coupling constants in hertz (Hz) were measured from one-dimensional spectra, and multiplicities were abbreviated as follows: br (broad), s (singlet), d (doublet), t (triplet), and m (multiplet). The chemical shifts were recorded in ppm. The (C, H, N) microanalyses were performed on a Flash EA 1112 CHNS-O/MAS (CHN Analyzer) instrument (Thermo Finnigan, Italy). Sulfur was determined by complexometric titration with Chlorasenazo III. Fluorine content was determined on a SPECOL 11 (Carl Zeiss Jena, Germany) spectrophotometer. Melting points (uncorrected) were determined with SMP50 Stuart Automatic melting point (Cole-Palmer Ltd. Stone, Staffordshire, UK).

Synthesis of ethynylpyrroles 4a–k, ethynylindole 6, ethynylfuran 8. General procedure

Acylethynylpyrrole **1a–k**, 3-acylethynylindole **5** or 2-acylethynylfuran **7** (1 mmol) was dissolved in dry THF/MeCN (1:1, 4 mL), and then *t*-BuOK (224 mg, 2 mmol) was added to reaction mixture under nitrogen. Reaction mixture was stirred at room temperature for 1 hour while turning into an orange suspension. Then reaction mixture was diluted with cold (0-5 °C) water (30 mL) and extracted by cold (0-5 °C) *n*-hexane (3×10 ml). Combined extracts were washed with water (3×5 mL) and dried over Na_2SO_4. The residue, after removing solvent, was purified by flash chromatography (dried SiO_2, *n*-hexane) to afford ethynylpyrrole **4a–k,** ethynylindole **6** and ethynylfuran **8**.

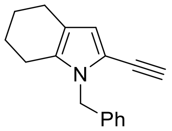

1-Benzyl-2-ethynyl-4,5,6,7-tetrahydro-1*H*-indole (4a). Yield: 197 mg (84%), colorless oil; ^1H NMR (400.13 MHz, $CDCl_3$): δ 7.37–7.24 (m, 3H, H*m,p,* Ph), 7.13–7.08 (m, 2H, H*o,* Ph), 6.37 (s, 1H, H-3, pyrrole), 5.14 (s, 2H, CH_2-Ph), 3.35 (s, 1H, ≡CH), 2.54–2.49 (m, 2H, CH_2-7), 2.43–2.38 (m, 2H, CH_2-4), 1.82–1.68 (m, 4H, CH_2-5, CH_2-6); ^{13}C NMR (100.6 MHz, $CDCl_3$): δ 138.3, 130.9, 128.7 (2C), 127.3, 126.7 (2C), 118.0, 114.2, 99.7, 81.2, 77.0, 47.9, 23.5, 23.2, 23.1, 22.5; IR (KBr) 3287, 3087, 3063, 3030, 2928, 2849, 2097, 1495, 1457, 1388, 1357, 1301, 1130, 1077, 1029, 928, 795, 722, 696, 545, 457 cm^{-1}; Anal. Calcd for $C_{17}H_{17}N$: C, 86.77; H, 7.28; N, 5.95%. Found: C, 86.47; H, 7.31; N, 6.14%.

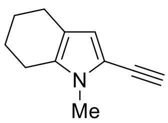

2-Ethynyl-1-methyl-4,5,6,7-tetrahydro-1*H*-indole (4b). Yield: 137 mg (86%), white crystals, mp 53-54 ºC; ^{1}H NMR (400.13 MHz, $CDCl_3$): δ 6.26 (s, 1H, H-3, pyrrole), 3.50 (s, 3H, NMe), 3.37 (s, 1H, ≡CH), 2.52–2.50 (m, 2H, CH_2-7), 2.47–2.45 (m, 2H, CH_2-4), 1.83–1.80 (m, 2H, CH_2-5), 1.73–1.71 (m, 2H, CH_2-6); ^{13}C NMR (100.6 MHz, $CDCl_3$,): δ 130.9, 117.4, 113.6, 112.6, 81.1, 76.9, 30.8, 23.6, 23.2, 23.0, 22.4; IR (film) 3288, 3100, 2929, 2847, 2097, 1570, 1462, 1442, 1386, 1302, 1130, 1055, 790, 667, 536 cm^{-1}; Anal. Calcd for $C_{11}H_{13}N$: C, 82.97; H, 8.23; N, 8.80%. Found: C, 82.71; H, 8.44; N, 8.58%.

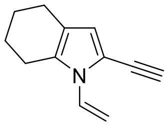

2-Ethynyl-1-vinyl-4,5,6,7-tetrahydro-1*H*-indole (4c). Yield: 127 mg (74%), colorless oil; ^{1}H NMR (400.13 MHz, $CDCl_3$): δ 6.97 (dd, *J*=16.1, 9.4 Hz, 1H, H_x), 6.34 (s, 1H, H-3, pyrrole), 5.34 (d, *J*=16.1 Hz, 1H, H_a), 4.83 (d, *J*=9.4 Hz, 1H, H_b), 3.39 (s, 1H, ≡CH), 2.66–2.63 (m, 2H, CH_2-7), 2.48–2.45 (m, 2H, CH_2-4), 1.83–1.80 (m, 2H, CH_2-5), 1.71–1.69 (m, 2H, CH_2-6); ^{13}C NMR (100.6 MHz, $CDCl_3$): δ 130.5, 119.5, 116.8, 112.3, 102.1, 99.7, 82.1, 76.8, 24.2, 23.4, 23.1, 23.0; IR (film) 3292, 3128, 3049, 2932, 2849, 2099, 1643, 1577, 1483, 1438, 1387, 1324, 1294, 1136, 966, 871, 802, 669, 558 cm^{-1}; Anal. Calcd for $C_{12}H_{13}N$: C, 84.17; H, 7.65; N, 8.18%. Found: C, 83.85; H, 7.81; N, 8.36%.

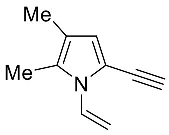

5-Ethynyl-2,3-dimethyl-1-vinyl-1*H*-pyrrole (4d). Yield: 52 mg (36%), colorless oil; ^{1}H NMR (400.13 MHz, $CDCl_3$): δ 6.91 (dd, *J* = 16.0, 9.2 Hz, 1H, H_x), 6.36 (s, 1H, H-3, pyrrole), 5.46 (d, *J*=16.1 Hz, 1H, H_a), 4.94 (d, *J* = 9.2 Hz, 1H, H_b), 3.37 (s, 1H, ≡CH), 2.21 (s, 3H, Me), 1.99 (s, 3H, Me); ^{13}C NMR (100.6 MHz, $CDCl_3$): δ 130.8, 127.8, 119.0, 116.7, 111.6, 104.5, 81.7, 76.9, 11.4, 11.1; IR (film) 3291, 3106, 2920, 2866, 2099, 1643, 1483, 1432, 1392, 1335, 1310, 1162, 1113, 965, 879, 806, 671, 562 cm^{-1}; Anal. Calcd for $C_{10}H_{11}N$: C, 82.72; H, 7.64; N, 9.65%. Found: C, 82.94; H, 7.49; N, 9.80%.

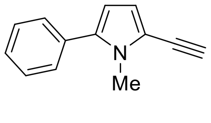

2-Ethynyl-1-methyl-5-phenyl-1*H*-pyrrole (4e). Yield: 172 mg (95%), colorless oil; ^{1}H NMR (400.13 MHz, $CDCl_3$): δ 7.42–7.34 (m, 5H, Ph), 6.55 (d, *J*=3.8 Hz, 1H, H-3, pyrrole), 6.16 (d, *J*=3.8 Hz, 1H, H-4, pyrrole), 3.69 (s, 3H, NMe), 3.44 (s, 1H, ≡CH); ^{13}C NMR (100.6 MHz, $CDCl_3$): δ 136.7, 132.9, 128.9 (2C),

128.6 (2C), 127.5, 116.1, 115.6, 108.6, 82.0, 76.5, 33.2; IR (film) 3287, 3106, 3060, 2948, 2102, 1602, 1498, 1457, 1390, 1324, 1234, 1155, 1074, 1028, 758, 698, 568 cm^{-1}; Anal. Calcd for $C_{13}H_{11}N$: C, 86.15; H, 6.12; N, 7.73%. Found: C, 85.75; H, 5.86; N, 7.48%.

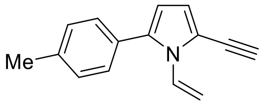

2-Ethynyl-5-(4-methylphenyl)-1-vinyl-1*H*-pyrrole (4f). Yield: 174 mg (84%), colorless oil; ^{1}H NMR (400.13 MHz, $CDCl_3$): δ 7.34–7.28 (m, 2H, H*o*, Ph), 7.24–7.17 (m, 2H, H*m*, Ph), 6.82 (dd, *J*=15.9, 9.0 Hz, 1H, H_x), 6.63 (d, *J*=3.8 Hz, 1H, H-3 pyrrole), 6.17 (d, *J*=3.8 Hz, 1H, H-4, pyrrole), 5.53 (d, *J*=15.9 Hz, 1H, H_a), 4.99 (d, *J*=9.0 Hz, 1H, H_b), 3.43 (s, 1H, ≡CH), 2.38 (s, 3H, Me); ^{13}C NMR (100.6 MHz, $CDCl_3$): δ 137.6, 136.1, 131.1, 129.6, 129.2 (2C), 129.1 (2C), 118.5, 114.5, 109.9, 107.0, 82.5, 76.8, 21.3; IR (KBr) 3287, 3112, 3024, 2921, 2102, 1643, 1547, 1510, 1466, 1419, 1389, 1324, 1297, 1226, 1113, 963, 889, 822, 775, 672, 571, 500 cm^{-1}; Anal. Calcd for $C_{15}H_{13}N$: C, 86.92; H, 6.32; N, 6.76%. Found: C, 86.68; H, 6.51; N, 6.85%.

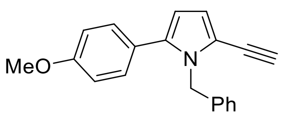

1-Benzyl-2-ethynyl-5-(4-methoxyphenyl)-1*H*-pyrrole (4g). Yield: 253 mg (88%), white crystals; mp 92-93°C; ^{1}H NMR (400.13 MHz, $CDCl_3$): δ 7.30–7.22 (m, 3H, H*m,p*, Ph), 7.20–7.15 (m, 2H, H*o*, Ph), 6.99–6.93 (m, 2H, H*m*, Ph), 6.87–6.82 (m, 2H, H*o*, Ph), 6.63 (d, *J*=3.7 Hz, 1H, H-3 pyrrole), 6.16 (d, *J*=3.7 Hz, 1H, H-4, pyrrole), 5.25 (s, 2H, CH_2-Ph), 3.80 (s, 3H, MeO), 3.29 (s, 1H, ≡CH); ^{13}C NMR ($CDCl_3$, 100.6 MHz): δ 159.3, 138.8, 136.7, 130.4 (2C), 128.6 (2C), 127.2, 126.3 (2C), 125.3, 116.1, 115.5, 114.0 (2C), 108.8, 81.8, 76.6, 55.4, 48.9; IR (KBr) 3287, 3087, 3063, 3031, 2955, 2934, 2836, 2100, 1611, 1575, 1547, 1510, 1463, 1442, 1392, 1358, 1321, 1288, 1249, 1178, 1110, 1087, 1031, 977, 909, 836, 767, 731, 695, 575, 524, 459 cm^{-1}; Anal. Calcd for $C_{20}H_{17}NO$: C, 83.59; H, 5.96; N, 4.87; O, 5.57%. Found: C, 83.31; H, 6.02; N, 5.02%.

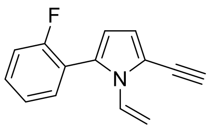

2-Ethynyl-5-(2-fluorophenyl)-1-vinyl-1*H*-pyrrole (4h). Yield: 192 mg (91%), colorless oil; ^{1}H NMR (400.13 MHz, $CDCl_3$): δ 7.40–7.30 (m, 2H, H*m*, Ph), 7.22–7.08 (m, 2H, H*o,p*, Ph), 6.84 (dd, *J*=15.9, 8.9 Hz, 1H, H_x), 6.66 (d, *J*=3.7 Hz, 1H, H-3 pyrrole), 6.24 (d, *J*=3.7 Hz, 1H, H-4, pyrrole), 5.34 (d, *J*=15.9 Hz, 1H, H_a), 4.91 (d, *J*=8.9 Hz, 1H, H_b), 3.45 (s, 1H, ≡CH); ^{13}C NMR (100.6 MHz, $CDCl_3$): δ 159.9 (d, *J*=249.1 Hz, C-2, 2-FC_6H_4), 132.1 (d, *J*=2.0 Hz, C-6, 2-FC_6H_4), 130.9, 130.1 (d, *J*=8.2 Hz, C-4, 2-FC_6H_4), 129.2, 124.24 (d, *J*=3.3 Hz, C-5, 2-FC_6H_4), 120.6 (d, *J*=15.5 Hz, C-1, 2-FC_6H_4), 118.1, 116.1 (d, *J*=22.0 Hz,

C-3, 2-FC_6H_4), 115.2, 111.8, 106.4, 82.7, 76.4; IR (KBr) 3293, 3115, 3068, 2924, 2104, 1645, 1580, 1547, 1498, 1465, 1397, 1300, 1229, 1109, 963, 890, 817, 780, 759, 672, 577, 471 cm^{-1}; Anal. Calcd for $C_{14}H_{10}FN$: C, 79.60; H, 4.77; F, 8.99; N, 6.63%. Found: C, 79.24; H, 4.96; F, 8.75; N, 6.39%.

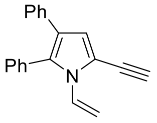

5-Ethynyl-2,3-diphenyl-1-vinyl-1*H*-pyrrole (4i). Yield: 242 mg (90%), white crystals; mp 93-94°C; ^{1}H NMR (400.13 MHz, $CDCl_3$): δ 7.39-7.34 (m, 3H, H*o,p*, Ph), 7.31–7.26 (m, 2H, H*o*, Ph), 7.21–7.15 (m, 2H, H*m*, Ph), 7.15–7.09 (m, 3H, H*m,p*, Ph), 6.84 (s, 1H, H-3 pyrrole), 6.71 (dd, *J*=15.9, 9.2 Hz, 1H, H_x), 5.47 (d, *J*=15.9 Hz, 1H, H_a), 4.91 (d, *J*=9.2 Hz, 1H, H_b), 3.46 (s, 1H, ≡CH); ^{13}C NMR (100.6 MHz, $CDCl_3$): δ 135.1, 131.9, 131.8, 131.4 (2C), 130.8, 128.7 (2C), 128.3 (2C), 128.2 (3C), 126.1, 123.8, 118.6, 113.7, 106.5, 82.8, 76.5; IR (KBr) 3274, 3080, 3057, 2923, 2100, 1641, 1601, 1557, 1495, 1446, 1386, 1320, 1305, 1177, 1031, 964, 889, 800, 769, 699, 587, 522 cm^{-1}; Anal. Calcd for $C_{20}H_{15}N$: C, 89.19; H, 5.61; N, 5.20%. Found: C, 88.89; H, 5.45; N, 5.34%.

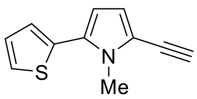

2-Ethynyl-1-methyl-5-(thiophen-2-yl)-1*H*-pyrrole (4j). Yield: 174 mg (93%), colorless oil; ^{1}H NMR (400.13 MHz, $CDCl_3$): δ 7.32–7.28 (m, 1H, H-5, thiophene), 7.10–7.05 (m, 2H, H-3,4, thiophene), 6.51 (d, *J*=3.9 Hz, 1H, H-3 pyrrole), 6.26 (d, *J*=3.9 Hz, 1H, H-4, pyrrole), 3.76 (s, 3H, N-CH_3), 3.43 (s, 1H, ≡CH); ^{13}C NMR (100.6 MHz, $CDCl_3$): δ 134.4, 129.2, 127.5, 125.8, 125.3, 116.6, 115.6, 109.7, 99.7, 82.2, 33.2; IR (KBr) 3288, 3106, 3074, 2944, 2922, 2101, 1445, 1417, 1395, 1345, 1314, 1201, 1034, 845, 766, 698, 570, 493 cm^{-1}; Anal. Calcd for $C_{11}H_9NS$: C, 70.55; H, 4.84; N, 7.48; S, 17.12%. Found: C, 70.26; H, 4.69; N, 7.28; S, 16.82%.

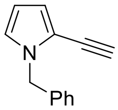

1-Benzyl-2-ethynyl-1*H*-pyrrole (4k). Yield: 145 mg (80%), colorless oil; ^{1}H NMR (400.13 MHz, $CDCl_3$): δ 7.36–7.27 (m, 3H, H*m,p*, Ph), 7.16–7.14 (m, 2H, H*o*, Ph), 6.68–6.65 (m, 1H, H-3, pyrrole), 6.54–6.51 (m, 1H, H-5, pyrrole), 6.13–6.10 (m, 1H, H-4, pyrrole), 5.19 (s, 2H, CH_2-Ph), 3.33 (s, 1H, ≡CH); ^{13}C NMR ($CDCl_3$, 100.6 MHz): δ 137.9, 128.8 (2C), 127.7, 127.3 (2C), 123.1, 116.0, 114.7, 108.7, 81.7, 76.0, 51.3; IR (KBr) 3288, 3106, 3064, 3031, 2925, 2853, 2103, 1495, 1466, 1455, 1435, 1300, 1018, 722, 694, 569, 522 cm^{-1}; Anal. Calcd for $C_{13}H_{11}N$: C, 86.15; H, 6.12; N, 7.73%. Found: C, 85.84; H, 5.89; N, 7.45%.

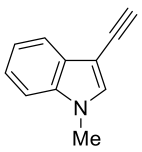

3-Ethynyl-1-methyl-1*H*-indole (6)

Yield: 113 mg (73%);

Spectral characteristics are the same as previously published [1].

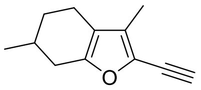

2-Ethynyl-3,6-dimethyl-4,5,6,7-tetrahydrobenzofuran (8)

Yield: 139 mg (80%), colorless oil; ^{1}H NMR (400.13 MHz, $CDCl_3$): δ 3.55 (s, 1H, ≡CH), 2.67–2.62 (m, 1H, CH), 2.33–2.30 (m, 2H, CH_2), 2.19–2.12 (m, 1H, CH), 2.00 (s, 3H, Me), 1.93–1.91 (m, 1H, CH), 1.85–1.81 (m, 1H, CH), 1.36–1.30 (m, 1H, CH), 1.07 (d, *J*=6.7 Hz, 3H, CHMe); ^{13}C NMR (100.6 MHz, $CDCl_3$): δ 152.1, 131.5, 127.2, 118.4, 83.8, 74.7, 31.7, 31.2, 29.6, 21.5, 20.0, 9.0; IR (KBr) 3293, 2923, 2849, 2103, 1628, 1558, 1456, 1379, 1295, 1257, 1150, 1107, 1066, 1041, 774, 692 cm^{-1}; Anal. Calcd for $C_{12}H_{14}O$: C, 82.72; H, 8.10; O, 9.18%. Found: C, 82.94; H, 7.88%.

Synthesis of propargyl alcohols 3a,c,d,f

Acylethynylpyrrole **1a,c,d,f** (1 mmol) was dissolved in dry MeCN (4 mL), and then *t*-BuONa (192 mg, 2 mmol) was added to reaction mixture under nitrogen and reaction mixture was stirred at room temperature for 1 hour. Then reaction mixture was diluted with water (30 mL) and extracted by diethyl ether (3×10 mL). Extracts were washed with water (3×5 mL). and dried over Na_2SO_4. The residue after removing solvents was fractionated by column chromatography (SiO_2, *n*-hexane : diethyl ether, 10 : 1) to afford propargyl alcohol **3a,c,d,f**.

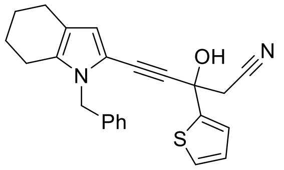

5-(1-Benzyl-4,5,6,7-tetrahydro-1*H*-indol-2-yl)-3-hydroxy-3-(thiophen-2-yl)pent-4-ynenitrile (3a). Spectral characteristics are the same as previously published [2].

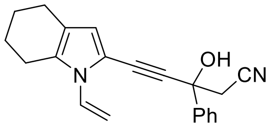

3-Hydroxy-3-phenyl-5-(1-vinyl-4,5,6,7-tetrahydro-1*H*-indol-2-yl)pent-4-ynenitrile (**3c**).

Yield: 224 mg (71%), yellow oil; ^{1}H NMR (400.13 MHz, $CDCl_3$): δ 7.72–7.71 (m, 2H, H*o*, Ph), 7.44–7.37 (m, 3H, H*m*,*p*, Ph), 6.98 (dd, *J* = 15.9, 9.3 Hz, 1H, H_x), 6.40 (s, 1H, H-3, pyrrole), 5.34 (d, *J* = 15.9 Hz, 1H, H_a), 4.88 (d, *J* = 9.3 Hz, 1H, H_b), 3.03 (d, *J* = 4.8 Hz, 2H, CH_2CN), 2.85 (s, 1H, OH), 2.67–2.65 (m, 2H, CH_2-7), 2.49–2.47 (m, 2H, CH_2-4), 1.83–1.81 (m, 2H, CH_2-5), 1.74–1.73 (m, 2H, CH_2-6); ^{13}C NMR ($CDCl_3$, 100.6 MHz): δ 141.7, 131.5, 130.4, 129.0, 128.8 (2C), 125.4 (2C), 119.9, 117.3, 116.4, 111.3, 103.2, 92.9, 81.4, 71.0, 35.7, 24.1, 23.3, 23.1, 23.0. IR (film) 3422, 3062, 3030, 2931, 2851, 2215, 1643, 1492, 1447, 1383, 1295, 1241, 1143, 1102, 1053, 968, 910, 805, 765, 733, 700, 646 cm^{-1}; Anal. Calcd for $C_{21}H_{20}N_2O$: C, 79.72; H, 6.37; N, 8.85; O, 5.06%. Found: C, 79.44; H, 6.20; N, 8.59%.

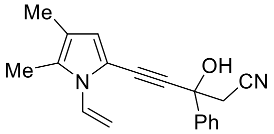

5-(4,5-Dimethyl-1-vinyl-1*H*-pyrrol-2-yl)-3-hydroxy-3-phenylpent-4-ynenitrile (3d).

Yield: 197 mg (68%), yellow crystals, mp 101-102 °C; ^{1}H NMR (400.13 MHz, $CDCl_3$): δ 7.72–7.70 (m, 2H, H*o*, Ph), 7.42–7.40 (m, 2H, H*m*,*p*, Ph), 6.91 (dd, *J* = 15.9, 9.1 Hz, 1H, H_x), 6.41 (s, 1H, H-3, pyrrole), 5.45 (d, *J* = 15.9 Hz, 1H, H_a), 4.99 (d, *J* = 9.1 Hz, 1H, H_b), 3.02 (d, *J* = 5.1 Hz, 2H, CH_2CN), 2.86 (s, 1H, OH), 2.22 (s, 3H, Me), 2.00 (s, 3H, Me); ^{13}C NMR (100.6 MHz, $CDCl_3$): δ 141.7, 130.6, 128.9, 128.7 (2C), 128.6, 125.4 (2C), 119.5, 117.0, 116.4, 110.6, 105.6, 92.6, 81.4, 70.9, 35.6, 11.3, 11.1; IR (KBr) 3422, 3062, 3030, 2921, 2215, 1643, 1493, 1449, 1392, 1357, 1304, 1172, 1100, 1049, 967, 910, 809, 765, 733, 700, 634 cm^{-1}; Anal. Calcd for $C_{19}H_{18}N_2O$: C, 78.59; H, 6.25; N, 9.65; O, 5.51%. Found: C, 78.22; H, 6.02; N, 9.42%.

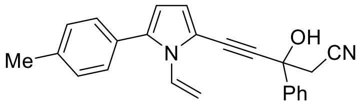

3-Hydroxy-3-phenyl-5-(5-(4-methylphenyl)-1-vinyl-1*H*-pyrrol-2-yl)pent-4-ynenitrile (3f).

Yield: 281 mg (80%), yellow oil; ^{1}H NMR ($CDCl_3$, 400 MHz): δ 7.74–7.72 (m, 2H, H*o*, Ph), 7.45–7.39 (m, 2H, H*m*,*p*, Ph), 7.31 (d, *J* = 7.9 Hz, 2H, H*o*, C_6H_4), 7.21 (d, *J* = 7.9 Hz, 2H, H*m*, C_6H_4), 6.83 (dd, *J* = 15.8, 8.9 Hz, 1H, H_x), 6.67 (d, *J* = 3.8 Hz, 1H, H-4, pyrrole), 6.22 (d, *J* = 3.8 Hz, 1H, H-3, pyrrole), 5.52 (d, *J* = 15.8 Hz, 1H, H_a), 5.05 (d, *J* = 8.9 Hz, 1H, H_b), 3.06 (d, *J* = 5.0 Hz, 2H, CH_2CN), 2.85 (s, 1H, OH), 2.39 (s, 3H, Me); ^{13}C NMR ($CDCl_3$, 100.6 MHz): δ 141.6, 137.8, 136.8, 131.2, 129.4, 129.3 (2C), 129.1 (3C), 128.8 (2C), 125.4 (2C), 118.9, 116.3, 113.7, 110.1, 108.0, 93.1, 81.4, 71.0, 35.6, 21.4; IR (KBr) 3416, 3061, 3028, 2922, 2218, 1643, 1515, 1472, 1449, 1418, 1389, 1324, 1301, 1224, 1112, 1042, 964, 909, 823, 773, 733,701, 622, 503 cm^{-1}; Anal. Calcd for $C_{24}H_{20}N_2O$: C, 81.79; H, 5.72; N, 7.95; O, 4.54%. Found: C, 81.35; H, 5.60; N, 7.68%.

Quantum chemical calculations

In order to study the thermodynamic aspects of propargyl alcohol decomposition, quantum chemical calculations using the density functional theory (DFT) method were performed. The calculations involved molecular structures shown in Scheme S1, which are obtained from those shown in Scheme 5 of the main text by setting R^1 = Me, R^2 = R^3 = H, R^4 = Ph, and additionally allowing K to be substituted with H, Li and Na.

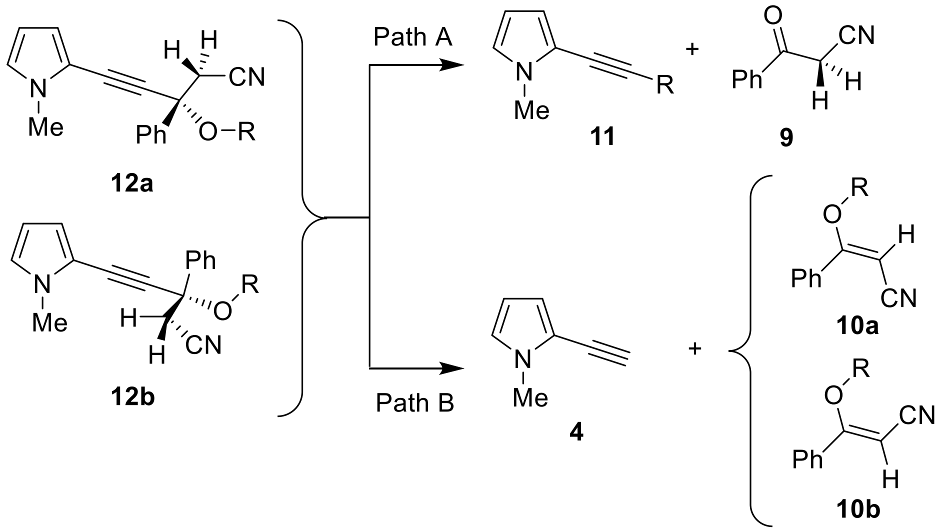

Scheme S1. Propargyl alcohol (R = H) and propargyl alcoholates (R = Li, Na, K) decomposition considered in the present quantum chemical study; molecular structures involved in the computations.

The focal point of the present study is to estimate the thermodynamic feasibility of path A and path B of the decomposition in Scheme S1. The Gibbs free energy change, ΔG, for each path was therefore evaluated. The Gibbs free energies, G, for each of the products and reactants, were obtained as sums of the total energy and the thermochemical correction (including all temperature-dependent contributions to G for T = 298.15 K) calculated using the B2PLYP [3] and B3LYP [4, 5] functionals, respectively, in combination with the 6-311G** basis set [6]. The molecular geometries were optimized at the B3LYP/6-311G** level of theory, and the solvent (acetonitrile) was treated at the level of the C-PCM model [7, 8]. This computational scheme (referred to as B2PLYP/6-311G**//B3LYP/6-311G** + C-PCM) proved to be very useful in our previous studies (see, e.g., Ref. [9], where also further details justifying its use could be found). All computations were performed using the Gaussian-09 suite of programs [10].

The major results of our calculations are summarized in Table S1, whereas the raw data obtained for the individual molecules are presented in Tables S2-S4.

Table S1. Gibbs free energy change (kJ/mol) for reaction paths A and B (scheme S1) and Gibbs free energy difference (kJ/mol) for conformers **12a** and **12b**(**12a-12b**) and isomers **10a** and **10b** (**10a-10b**) computed at the B2PLYP/6-311G**//B3LYP/6-311G** + C-PCM / acetonitrile level of theory. The results obtained for gas phase are shown for comparison, both for the B2PLYP/6-311G** (R = Li, K) and CBS-QB3 (R = Li) levels of theory.

R	Path A	Path B	**12a-12b**	**10a-10b**
H	-43.5	-15.1	1.3	7.9
Li	33.1	-66.5	-1.7	13.4
Na	18.4	-78.7	2.9	16.7
K	20.1	-88.3	-0.8	13.4
B2PLYP/6-311G**, gas phase				
Li	94.6	-78.7		
K	83.7	-84.5		
CBS-QB3, gas phase				
Li	77.8	-102.9		

According to our calculations, two conformations of the reactant, **12a** and **12b**, are possible (Fig. S1). Depending on the alkali metal, either one or the other is predicted to be more favorable (**12a** in the case of Li and K, and **12b** in the case of Na as well as in the case of R = H). The energy differences are, however, very small, the largest being only 2.9 kJ/mol, which means they are well within the accuracy margin of the method being used. We, therefore, consider further **12a** and **12b** as thermodynamically equally probable.

Similarly, the enolates formed in the reaction may have two isomeric forms with respect to the double bond, **10a** and **10b**. Here our calculations unambiguously predict the **10b** isomers to be more stable. Depending on the metal, it is expected to lie by 8-17 kJ/mol below the **10a** isomer.

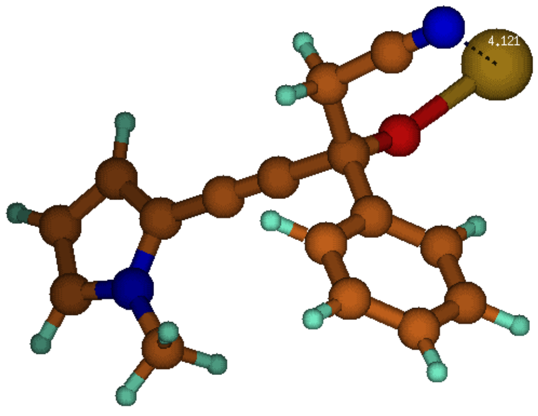

12a

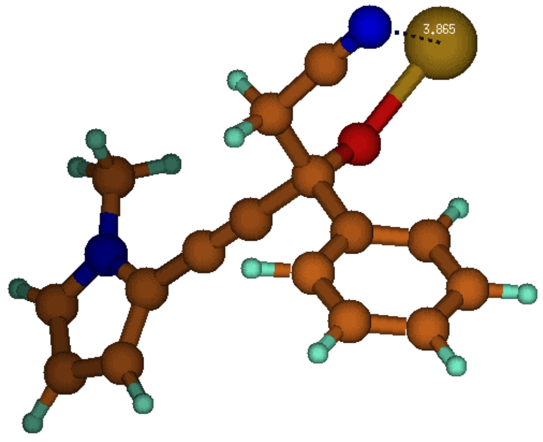

12b

Figure S1. Computed molecular structures of the reactant **12a** and **12b** in the case of potassium. (Note the length of the O-K bond, 4.12 and 3.87 Å, respectively).

The calculated ΔG values for the propargyl alcoholates decomposition reaction indicate that path A (i.e., formation of the metallated ethynylpyrroles and ketone) is thermodynamically closed, whereas path B (i.e., formation of the ethynylpyrrole and enolates) is open for all alkali metals considered. The exothermic

effect of the reaction in the latter case increases from Li (ΔG = –66.5 kJ/mol) to K (ΔG = –88.3 kJ/mol). In the case when no metal atoms are involved (R = H), both path A and path B are open; the former, however, is by 28.5 kJ/mol energetically more favorable.

In order to study the role of the solvent (acetonitrile), we have also performed calculations for the gas phase for R = Li, K (Table S1). As can be seen, path A remains closed, and path B remains open also in the gas phase. Interestingly, here the ΔG value for path B is roughly the same as in acetonitrile (the maximal change in the case of Li amounts to 13 kJ/mol) but is strongly increased for path A (by about 63 kJ/mol for both Li and K). This means that ketone **9** is stronger stabilized by acetonitrile than enolate **10b**. The latter result can be well explained by the polar character of acetonitrile and larger dipole moment of **9** compared to **10b** (according to our calculations, the gas phase μ values for R = H are 6.9 and 4.7 D, respectively).

Finally, the role of the improved electronic structure treatment was examined. To this end, the gas phase calculations for R = Li were performed at the level of the accurate composite complete basis set method CBS-QB3 [11, 12]. These shifts ΔG for path A and B down by 16.7 and 24.3 kJ/mol, respectively, compared to the gas phase B2PLYP/6-311G**//B3LYP/6-311G**treatment (Table S1) without qualitatively influencing the predictions of the latter scheme for the thermodynamics of the reaction under consideration.

Table S2. Results of the B2PLYP/6-311G**//B3LYP/6-311G** + C-PCM / acetonitrile calculations: total energy E (a.u.), thermochemical correction E_{TC} (a.u.) and Gibbs free energy G (a.u.) for molecular structures appearing in the reaction path A and path B (scheme S1, see text for details).

R	**12a**	**12b**	**11**	**9**	**10a**	**10b**
E						
H	-802.18270	-802.18255	-325.37972	-476.79758	-476.78465	-476.78732
Li	-809.18285	-809.18274	-332.35202		-483.80204	-483.80769
Na	-963.83078	-963.83051	-487.00423		-638.45390	-638.46134
K	-1401.43448	-1401.43443	-924.60666		-1076.05901	-1076.06696
E_{TC}						
H	0.21016	0.20962	0.08670	0.10118	0.10208	0.10179
Li	0.19692	0.19748	0.07515		0.08897	0.08949
Na	0.19484	0.19348	0.07065		0.08634	0.08740
K	0.19290	0.19322	0.06918		0.08203	0.08482
G						
H	-801.97254	-801.97294	-325.29302	-476.69641	-476.68257	-476.68554
Li	-808.98593	-808.98526	-332.27688		-483.71307	-483.71819
Na	-963.63594	-963.63703	-486.93358		-638.36756	-638.37394
K	-1401.24158	-1401.24121	-924.53747		-1075.97698	-1075.98214

Table S3. Results of the B2PLYP/6-311G** calculations for gas phase: total energy E (a.u.), thermochemical correction E_{TC} (a.u.) and Gibbs free energy G (a.u.) for molecular structures required for evaluation of ΔG of the reaction path A and path B for R = Li, K (scheme S1, see text for details). The molecular geometries are the same as in the B2PLYP/6-311G**//B3LYP/6-311G** + C-PCM / acetonitrile calculations.

	R	**12a**	**11**	**9**	**10b**
E					
	H		-325.37350	-476.78384	
	Li	-809.13404	-332.29040		-483.76823
	K	-1401.39456	-924.55510		-1076.03153
E_{TC}					
	H		0.08636	0.10074	
	Li	0.19998	0.07530		0.09120
	K	0.19462	0.07016		0.08659
G					
	H		-325.28714	-476.68310	
	Li	-808.93406	-332.21510		-483.67703
	K	-1401.19994	-924.48494		-1075.94494

Table S4. Results of the CBS-QB3 calculations for gas phase: total energy E (a.u.), thermochemical correction E_{TC} (a.u.) and Gibbs free energy G (a.u.) for molecular structures required for evaluation of ΔG of the reaction path A and path B for R = Li (scheme S1, see text for details). The molecular geometries are the same as in the B2PLYP/6-311G**//B3LYP/6-311G** + C-PCM / acetonitrile calculations.

	R	**12a**	**11**	**9**	**10b**
E					
	H		-325.71425	-477.24993	-484.25478
	Li	-809.95232	-332.64885		
E_{TC}					
	H		0.08636	0.10074	0.09119
	Li	0.19998	0.07530		
G					
	H		-325.62789	-477.14918	-484.16359
	Li	-809.75235	-332.57355		

NMR Spectra of synthesized compounds

^{1}H NMR spectrum of 1-benzyl-2-ethynyl-4,5,6,7-tetrahydro-1*H*-indole (**4a**) in $CDCl_3$

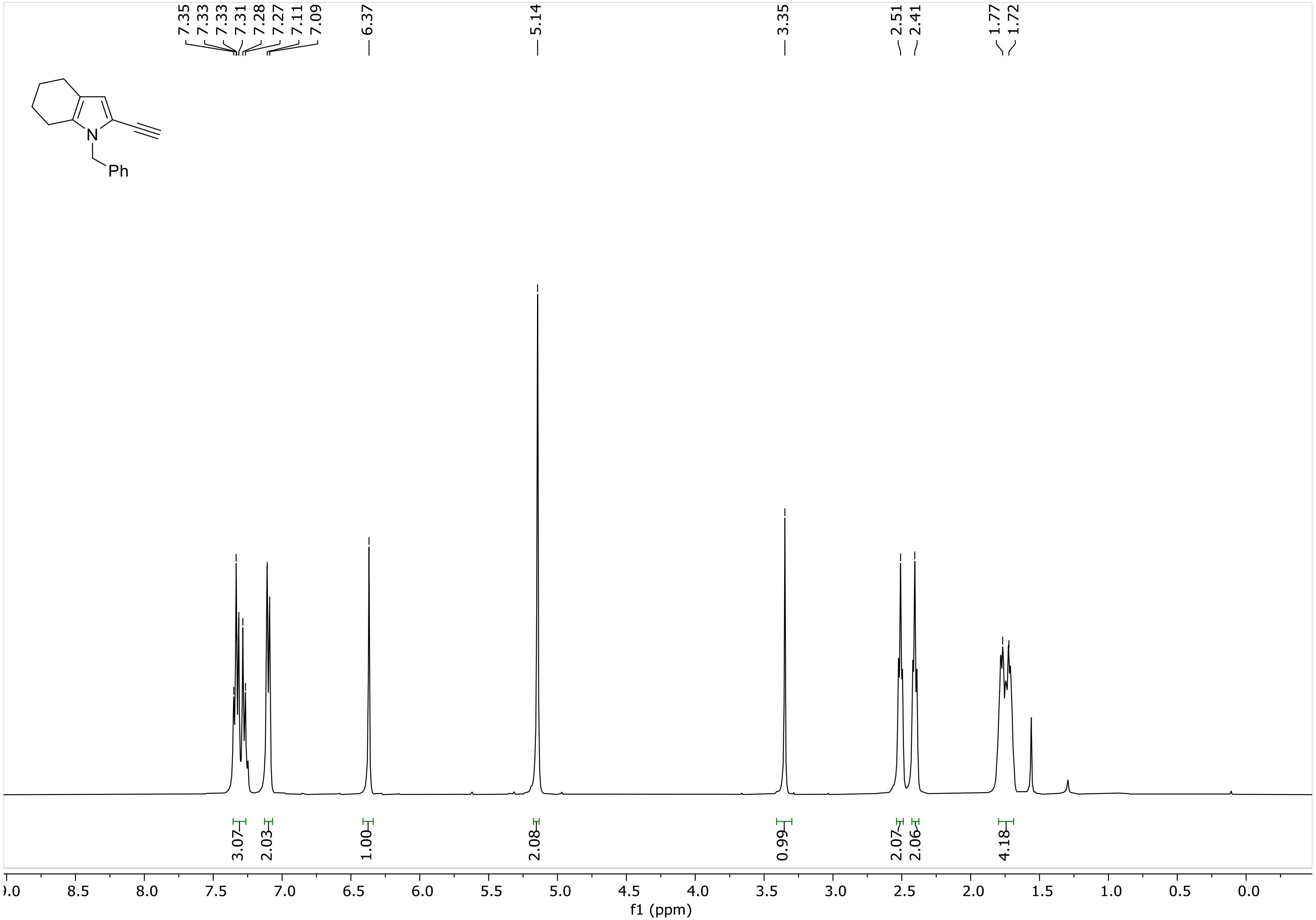

^{13}C NMR spectrum of 1-benzyl-2-ethynyl-4,5,6,7-tetrahydro-1*H*-indole (**4a**) in $CDCl_3$

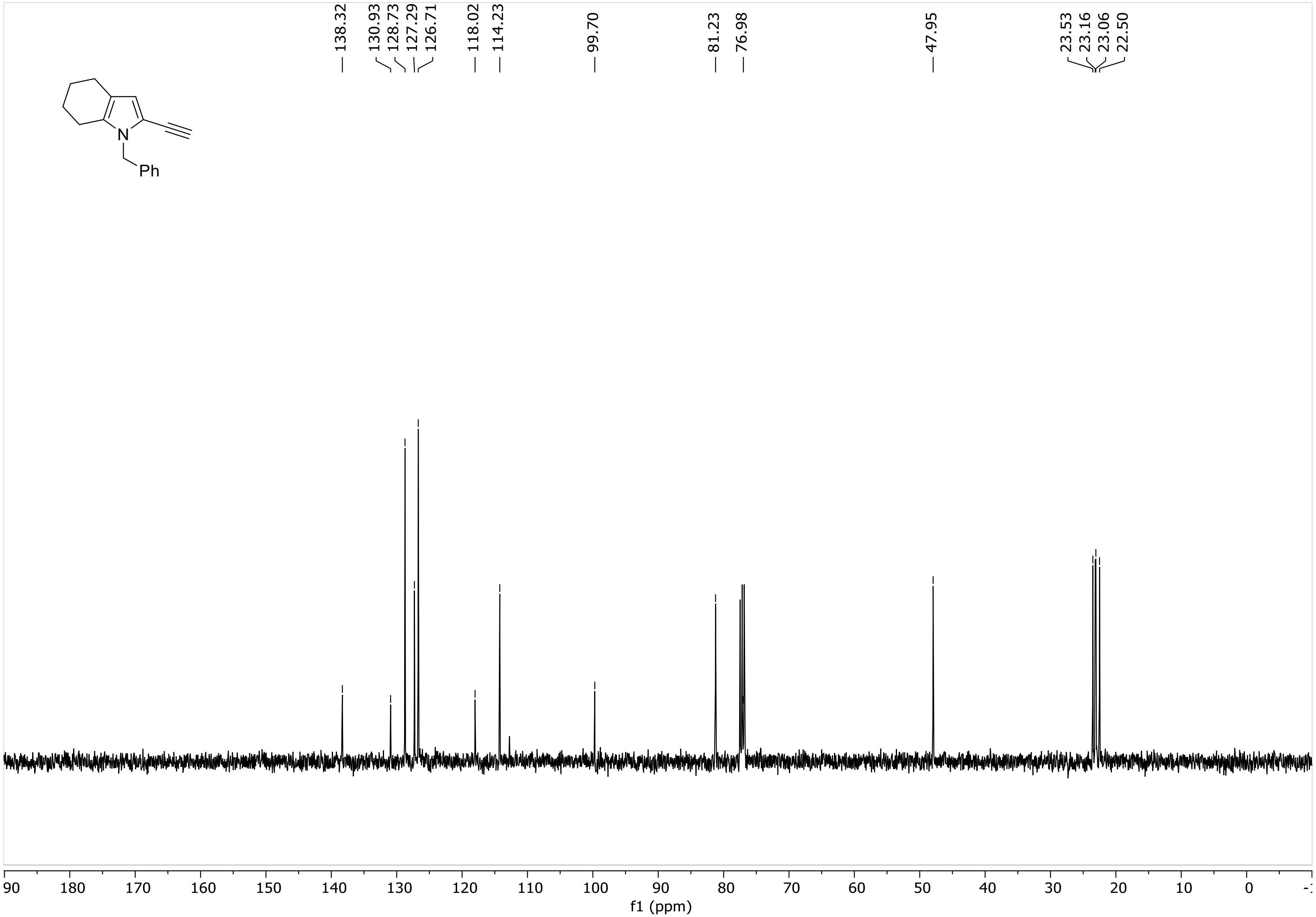

^1H NMR spectrum of 2-ethynyl-1-methyl-4,5,6,7-tetrahydro-1*H*-indole (**4b**) in $CDCl_3$

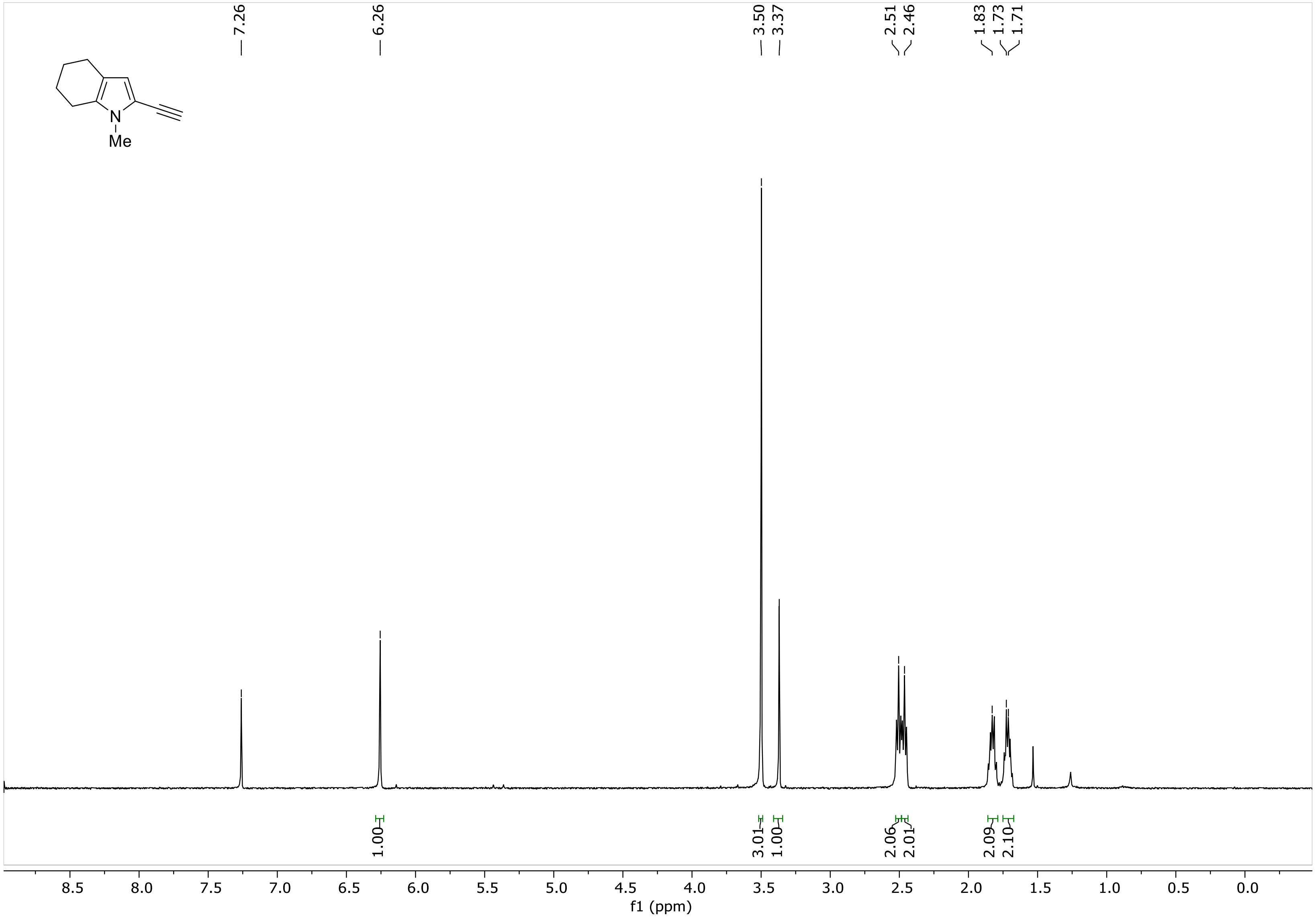

^{13}C NMR spectrum of 2-ethynyl-1-methyl-4,5,6,7-tetrahydro-1*H*-indole (**4b**) in $CDCl_3$

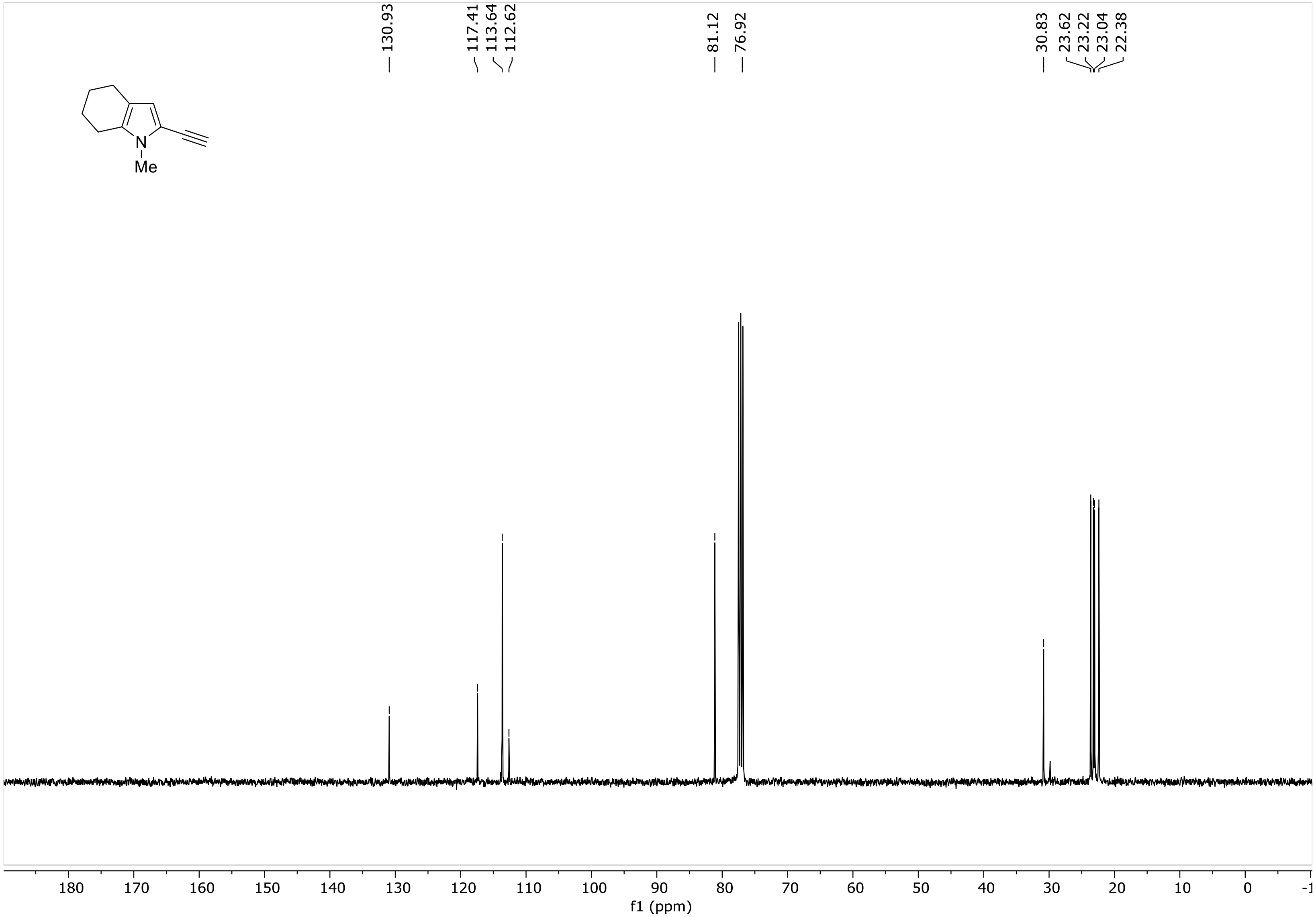

^{1}H NMR spectrum of 2-ethynyl-1-vinyl-4,5,6,7-tetrahydro-1*H*-indole (**4c**) in $CDCl_3$

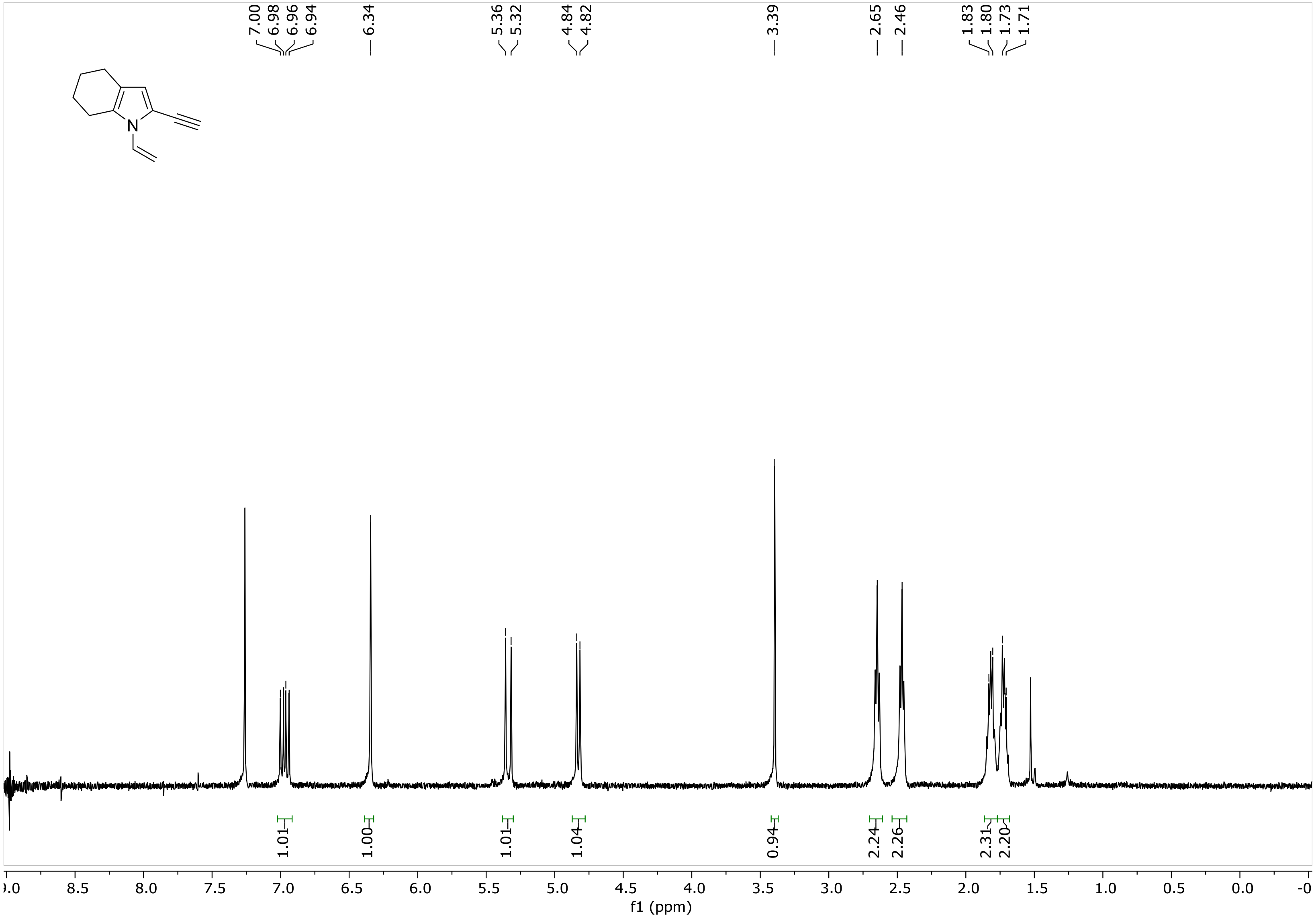

^{13}C NMR spectrum of 2-ethynyl-1-vinyl-4,5,6,7-tetrahydro-1*H*-indole (**4c**) in $CDCl_3$

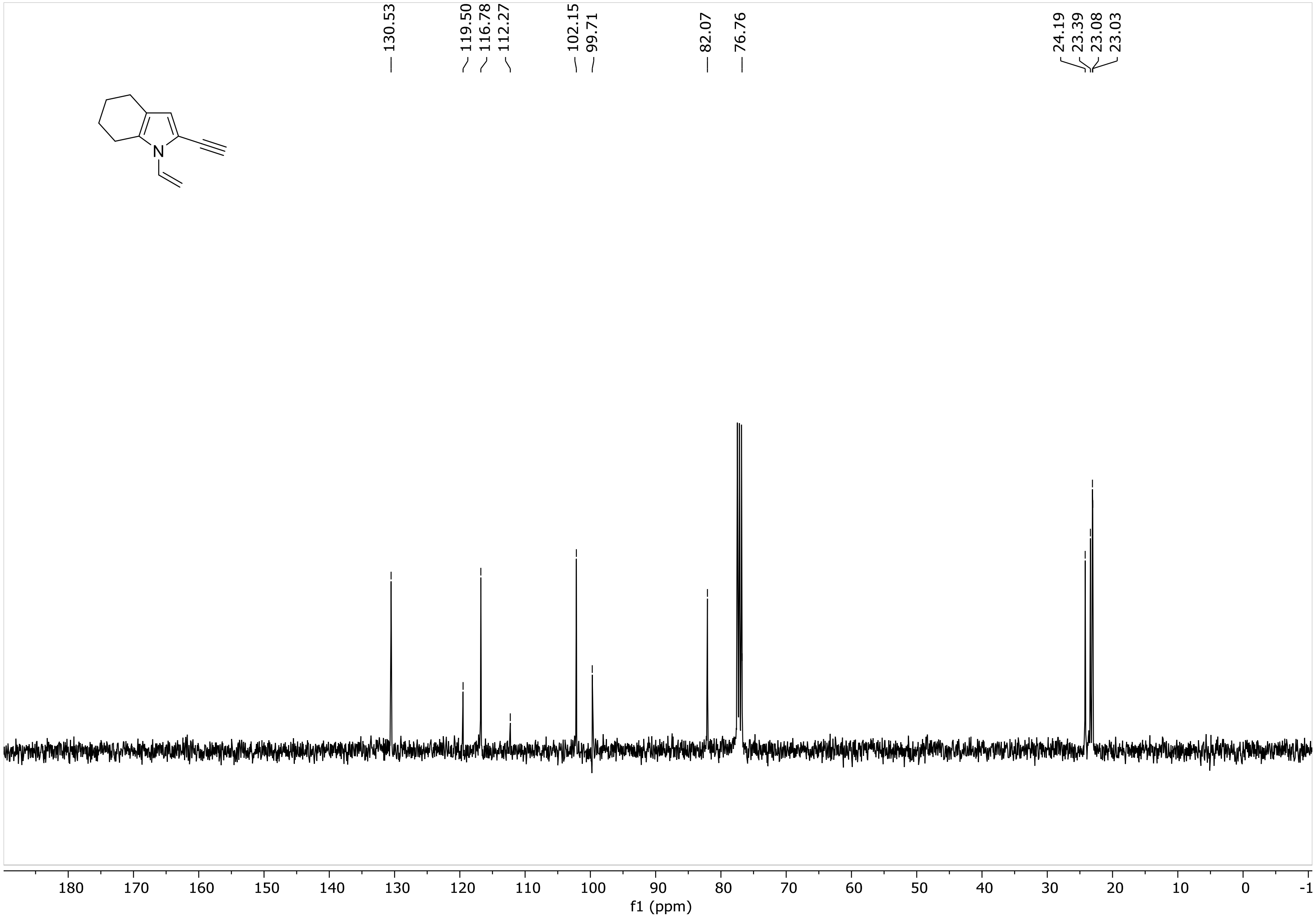

^{1}H NMR spectrum of 5-ethynyl-2,3-dimethyl-1-vinyl-1*H*-pyrrole (**4d**) in $CDCl_3$

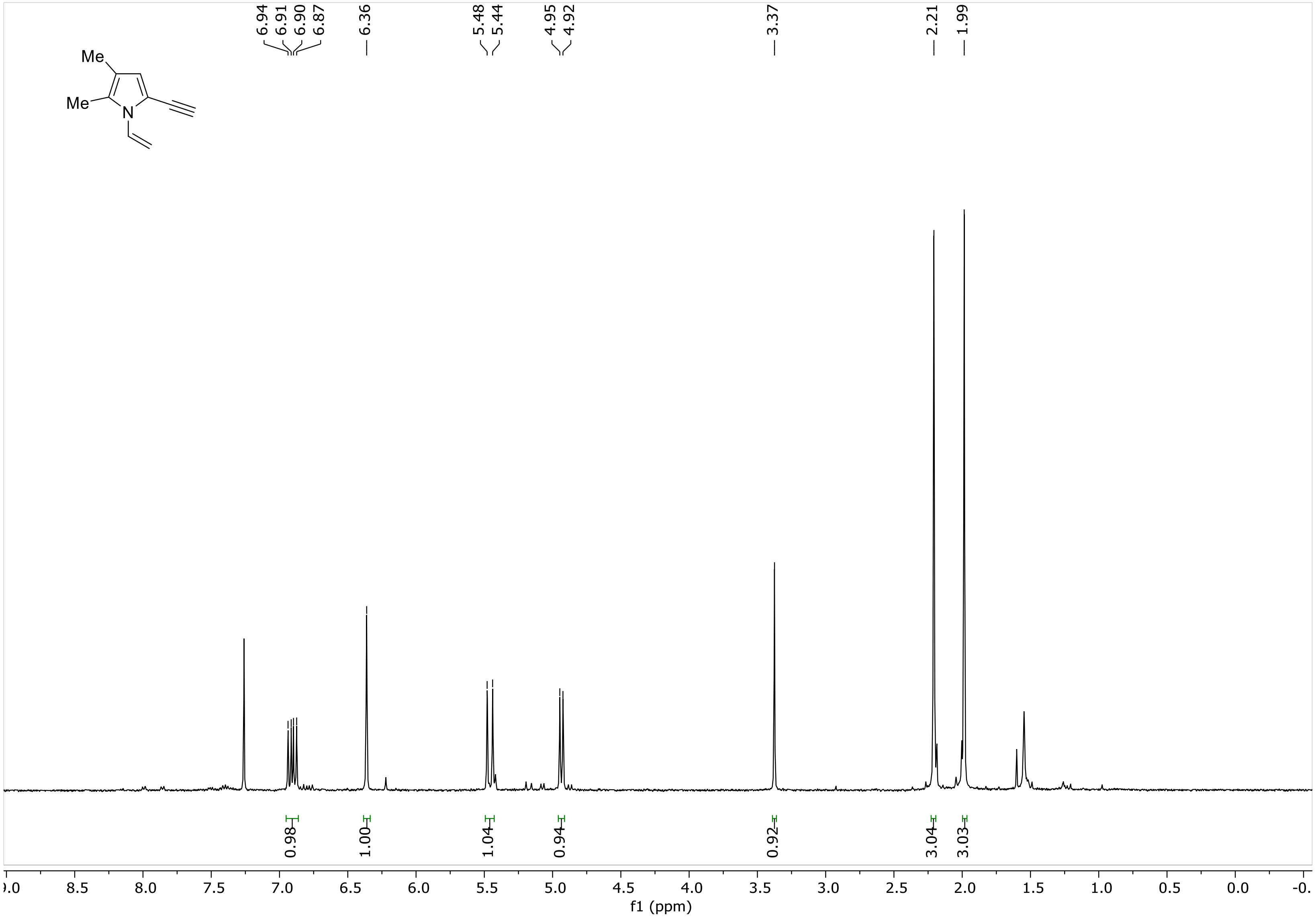

^{1}H NMR spectrum of 5-ethynyl-2,3-dimethyl-1-vinyl-1*H*-pyrrole (**4d**) in $CDCl_3$

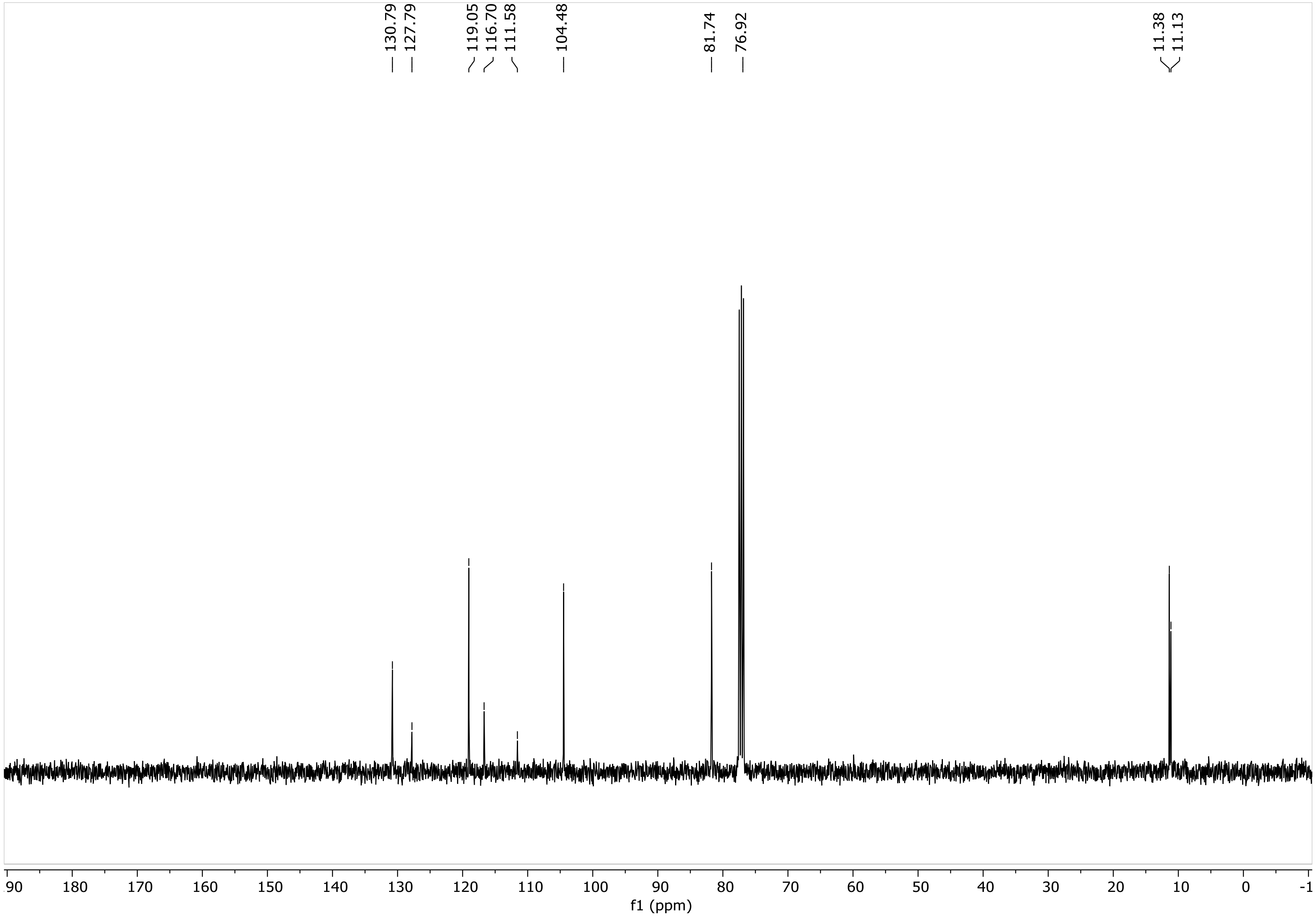

^{1}H NMR spectrum of 2-ethynyl-1-methyl-5-phenyl-1*H*-pyrrole (**4e**) in $CDCl_3$

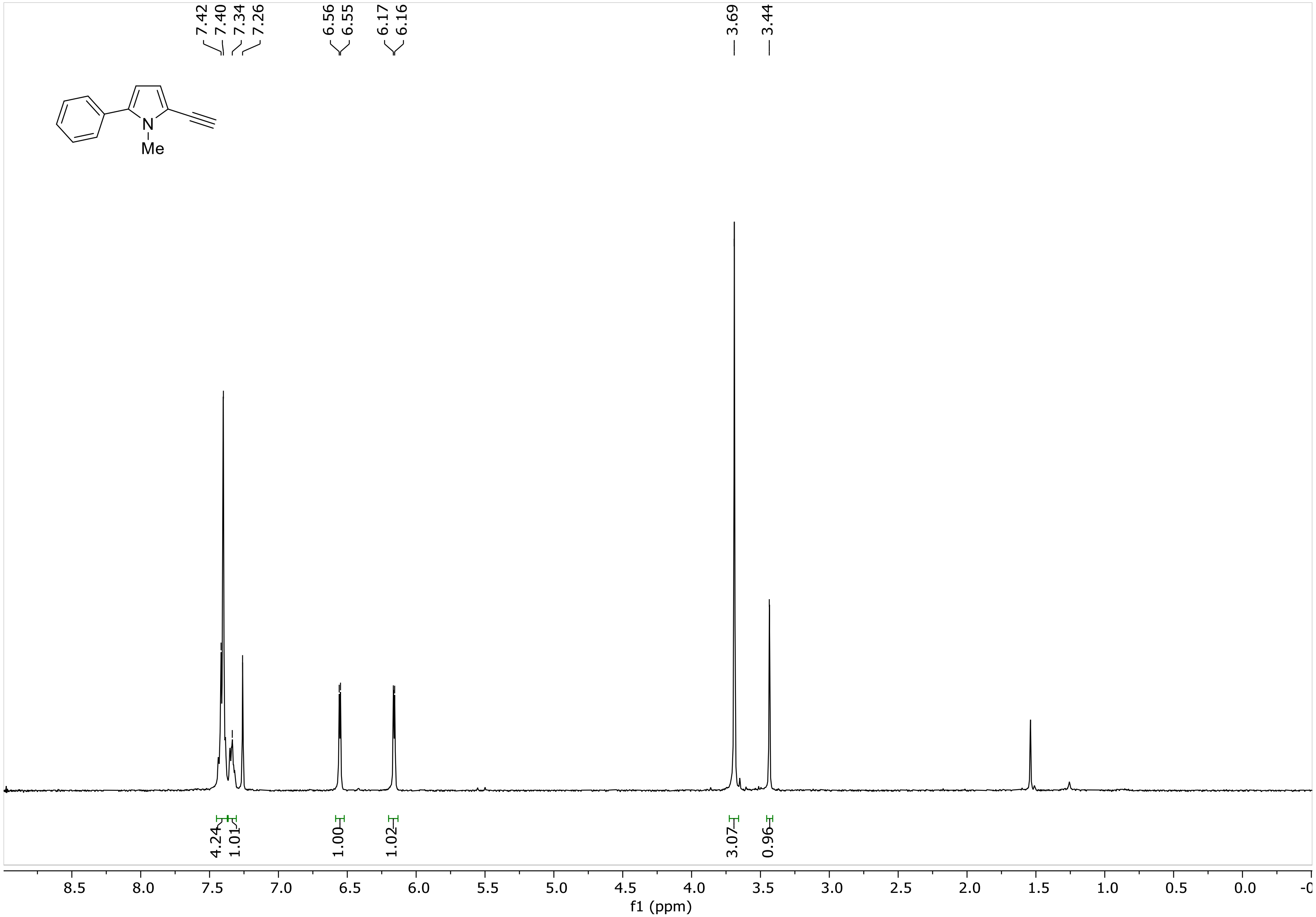

^{13}C NMR spectrum of 2-ethynyl-1-methyl-5-phenyl-1*H*-pyrrole (**4e**) in $CDCl_3$

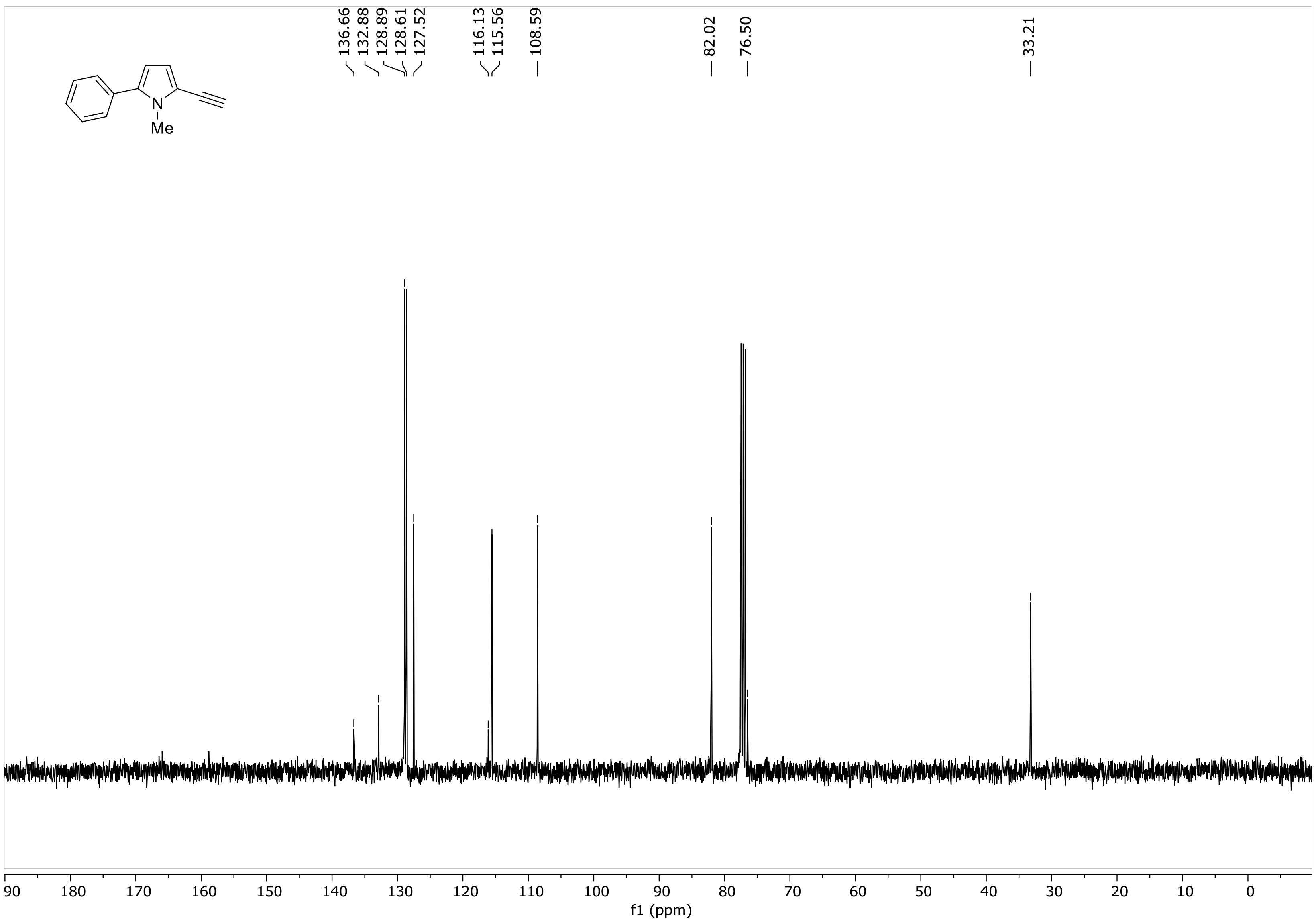

^{1}H NMR spectrum of 2-ethynyl-5-(p-tolyl)-1-vinyl-1*H*-pyrrole (**4f**) in $CDCl_3$

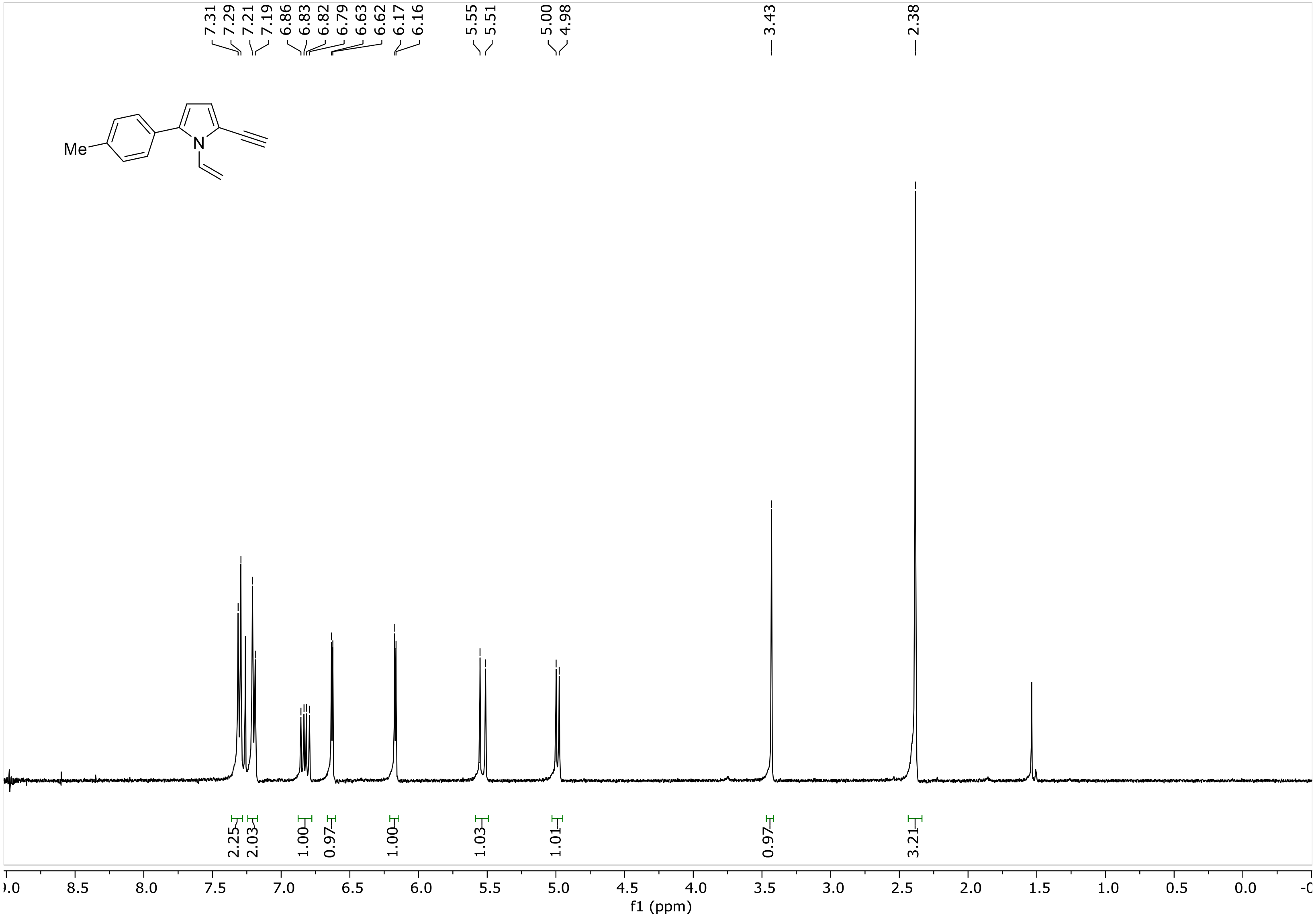

^{13}C NMR spectrum of 2-ethynyl-5-(*p*-tolyl)-1-vinyl-1*H*-pyrrole (**4f**) in $CDCl_3$

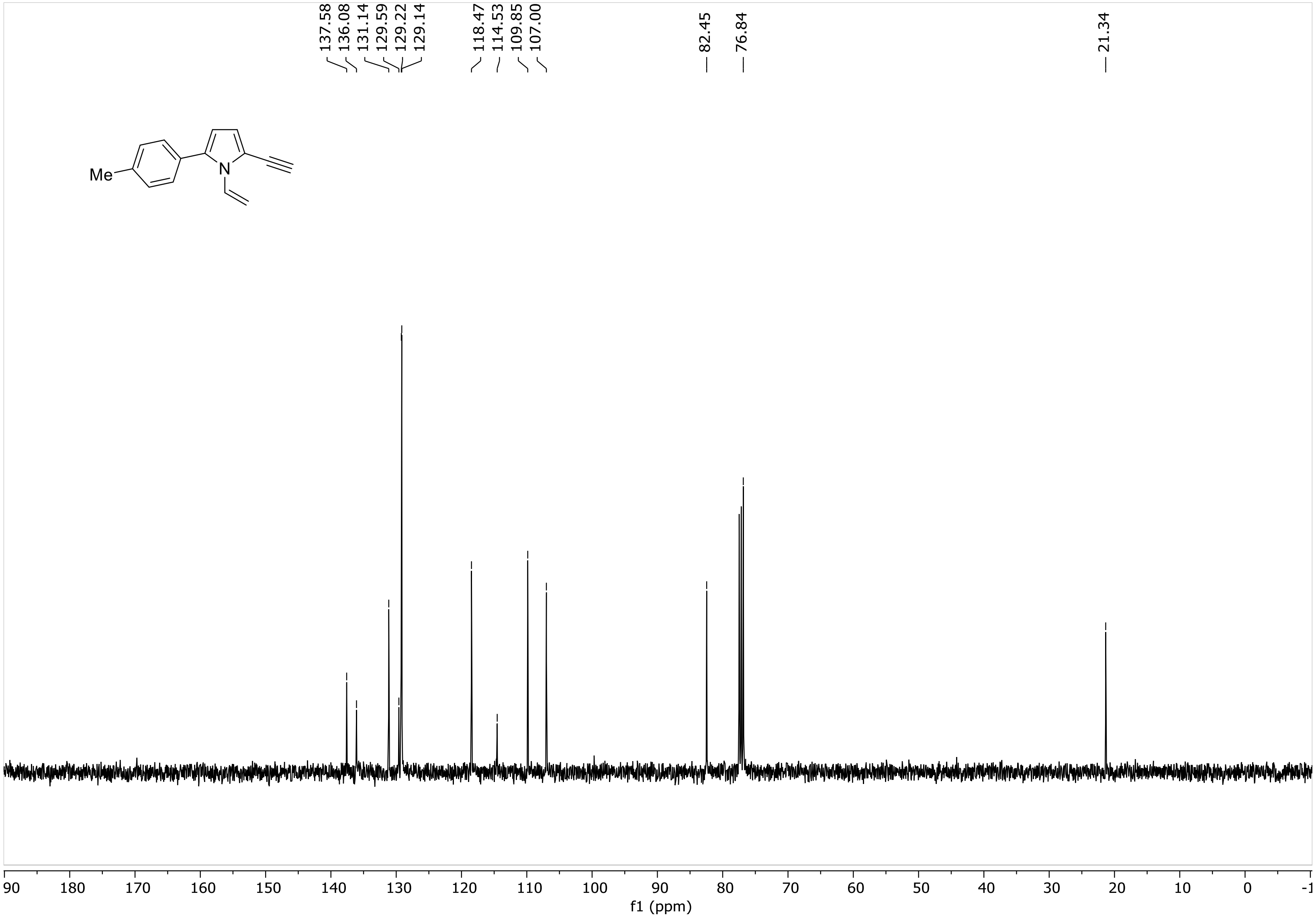

^{1}H NMR spectrum of 1-benzyl-2-ethynyl-5-(4-methoxyphenyl)-1*H*-pyrrole (**4g**) in $CDCl_3$

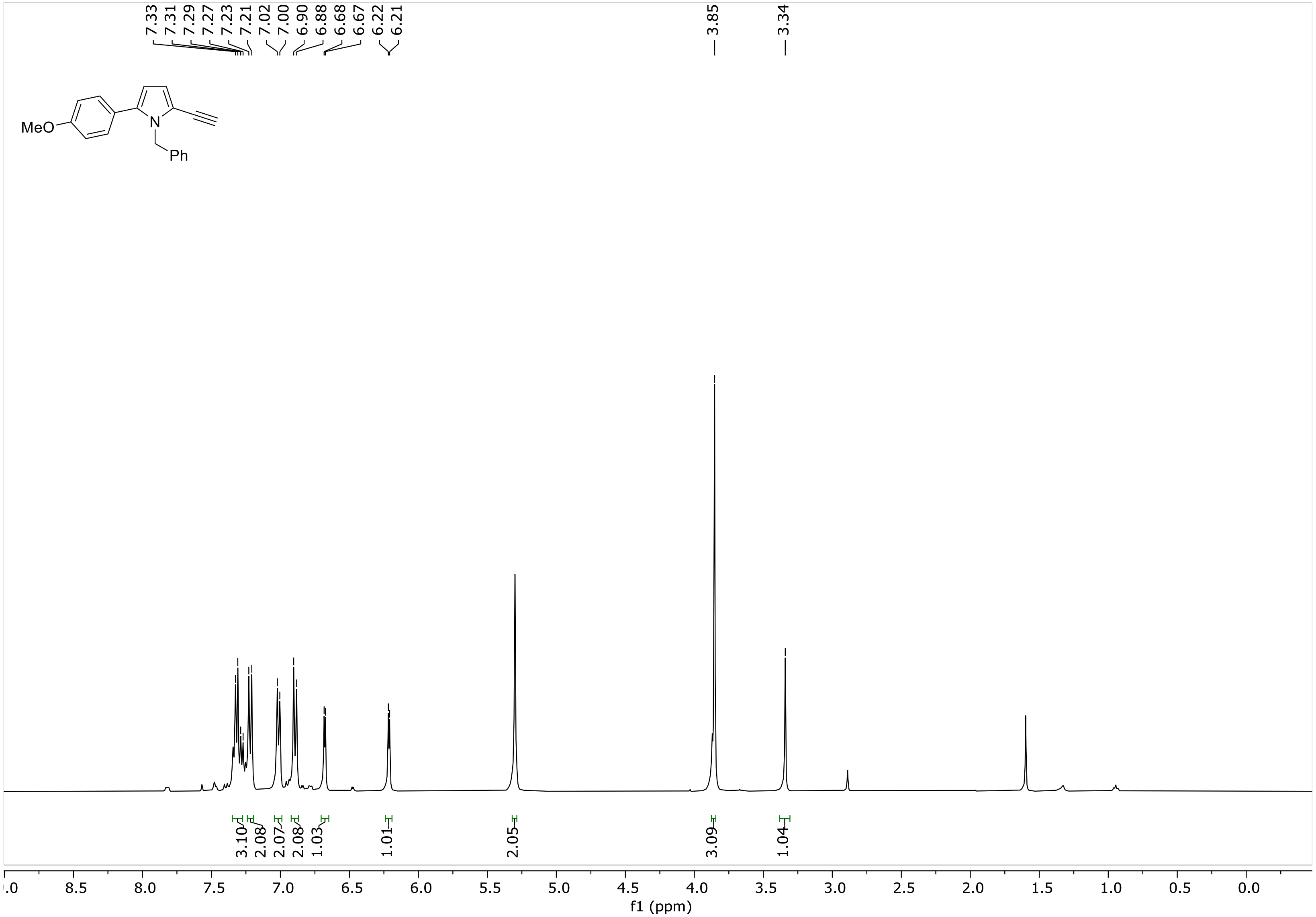

^{13}C NMR spectrum of 1-benzyl-2-ethynyl-5-(4-methoxyphenyl)-1*H*-pyrrole (**4g**) in $CDCl_3$

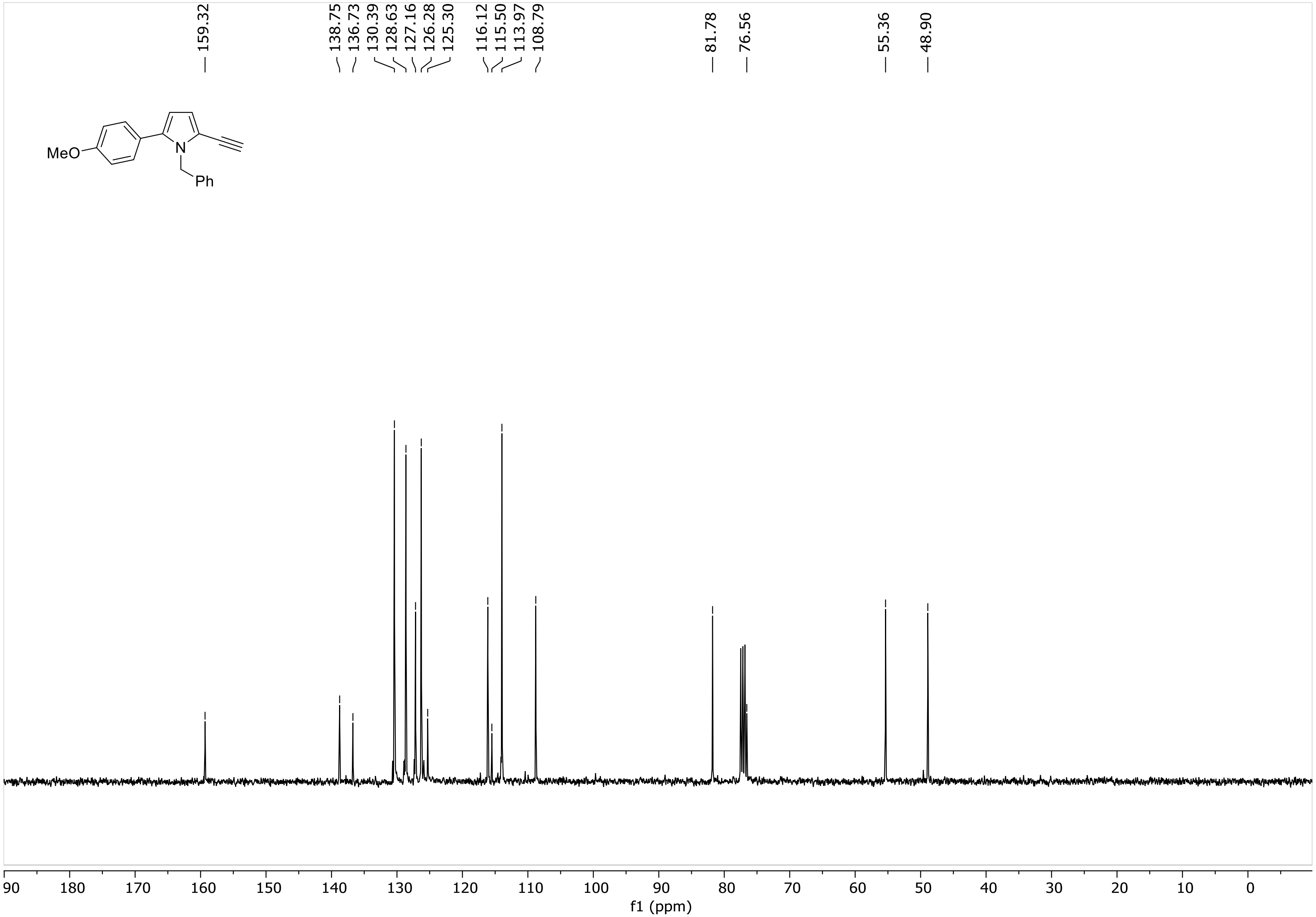

^{1}H NMR spectrum of 2-ethynyl-5-(2-fluorophenyl)-1-vinyl-1*H*-pyrrole (**4h**) in $CDCl_3$

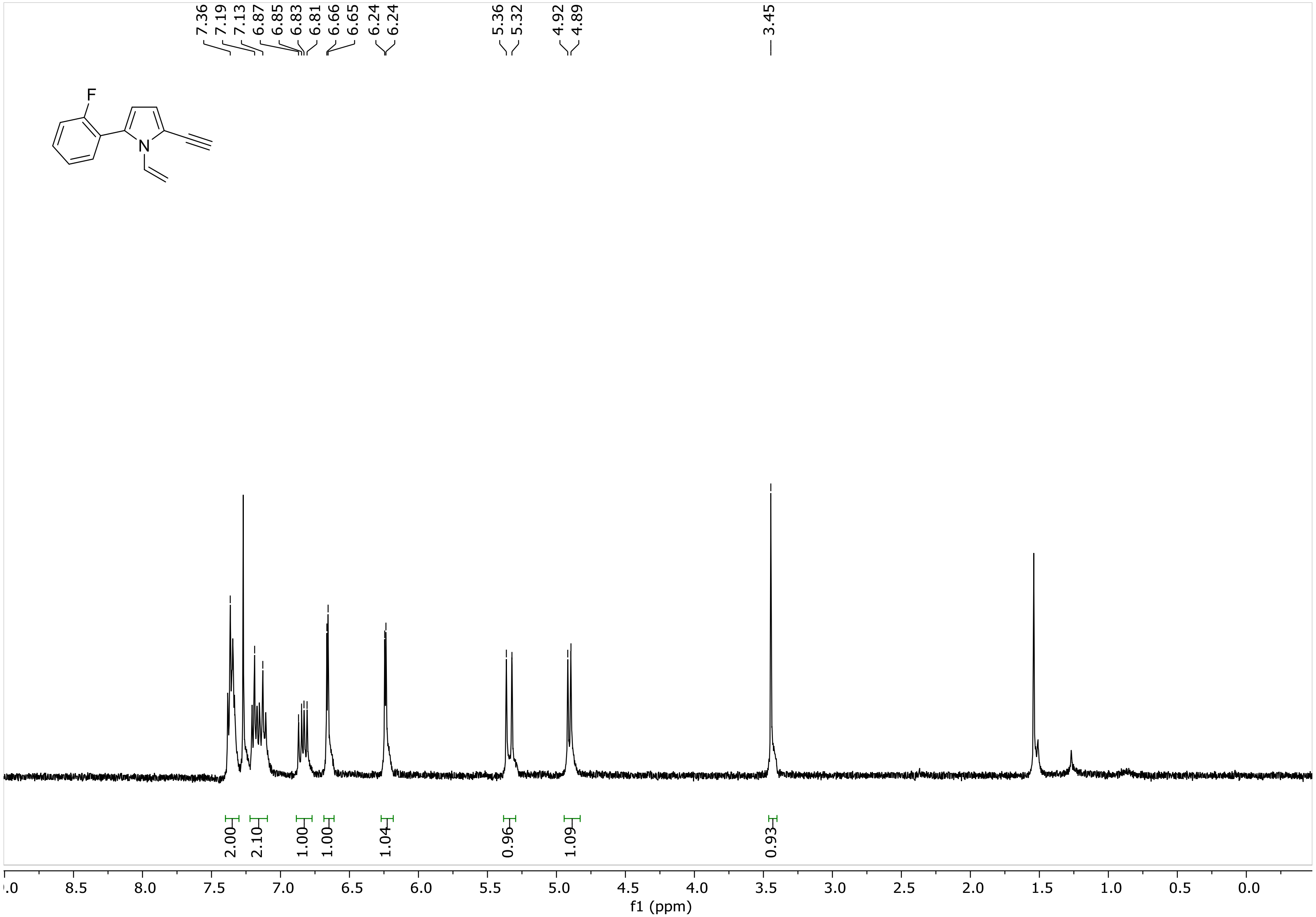

^{13}C NMR spectrum of 2-ethynyl-5-(2-fluorophenyl)-1-vinyl-1*H*-pyrrole (**4h**) in $CDCl_3$

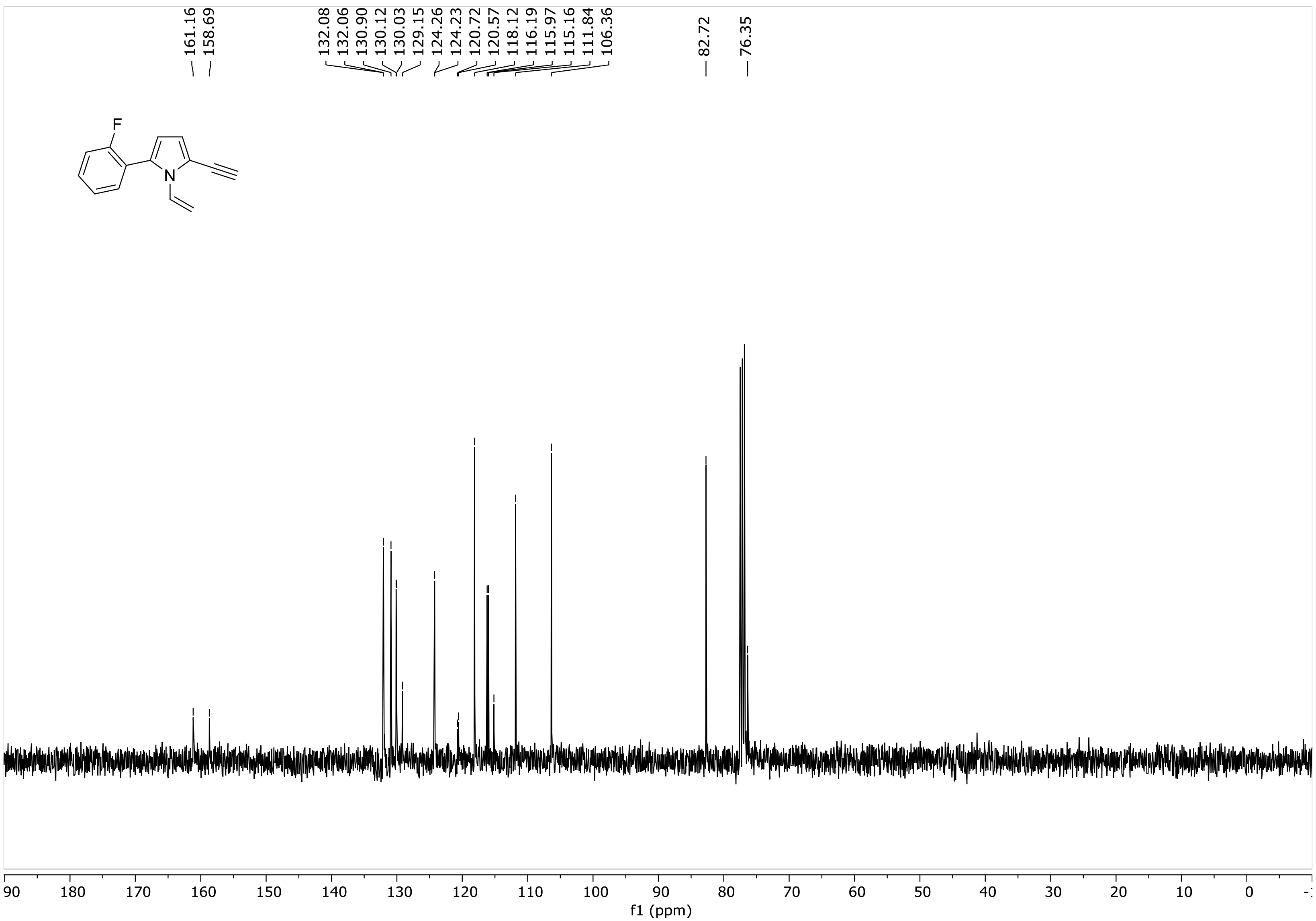

^{1}H NMR spectrum of 5-ethynyl-2,3-diphenyl-1-vinyl-1*H*-pyrrole (**4i**) in $CDCl_3$

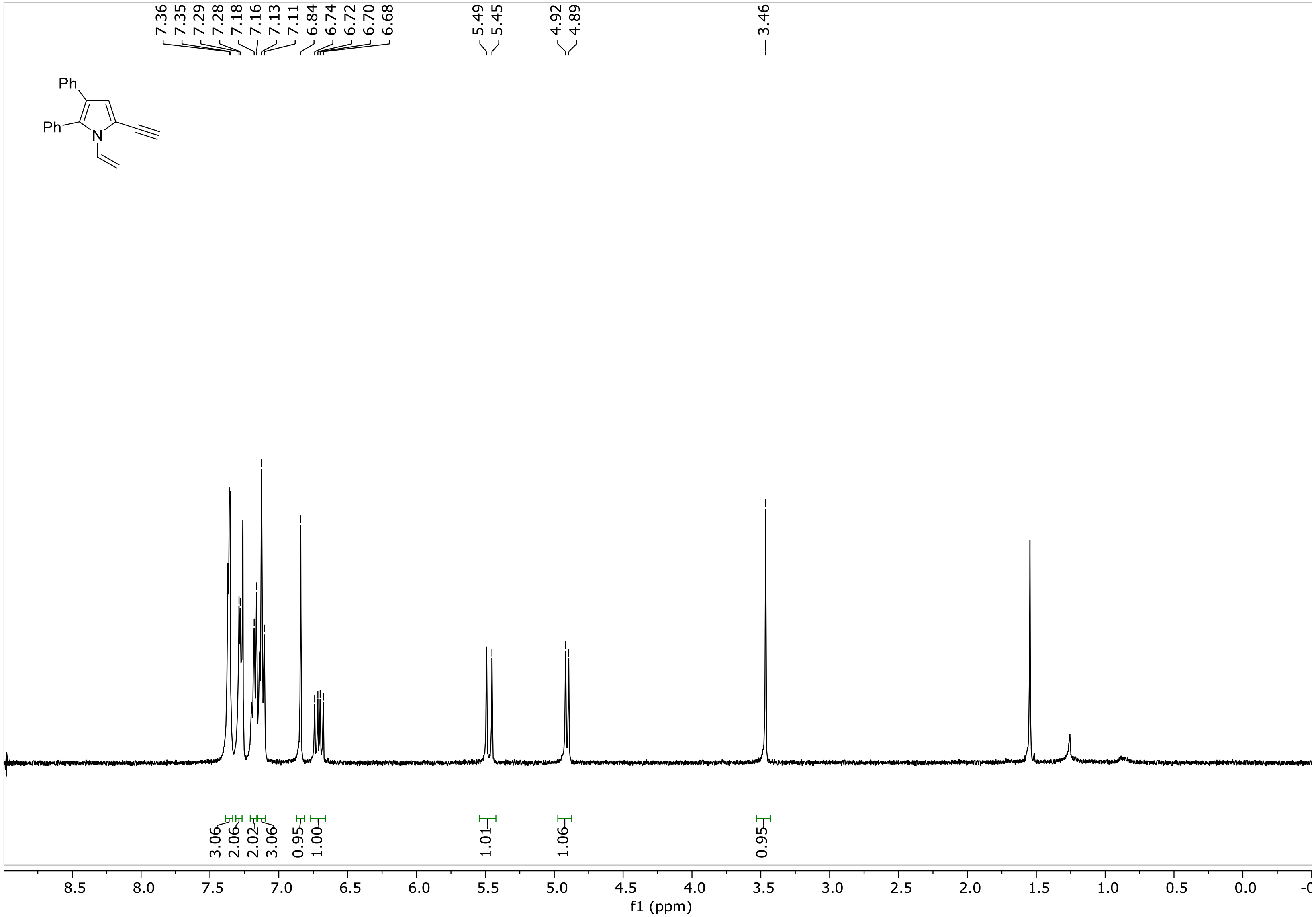

^{13}C NMR spectrum of 5-ethynyl-2,3-diphenyl-1-vinyl-1*H*-pyrrole (**4i**) in $CDCl_3$

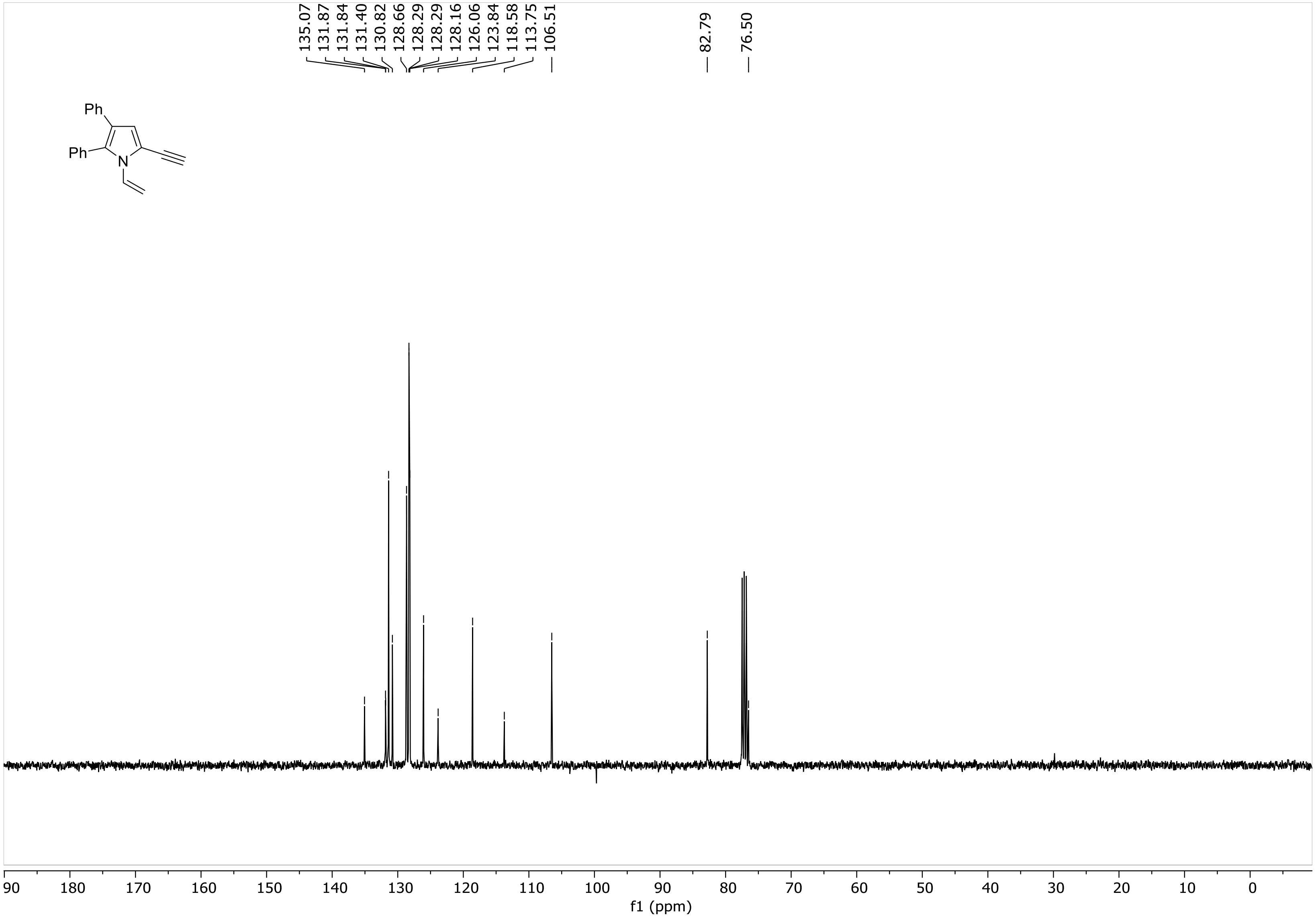

^{1}H NMR spectrum of 2-ethynyl-1-methyl-5-(thiophen-2-yl)-1*H*-pyrrole (**4j**) in $CDCl_3$

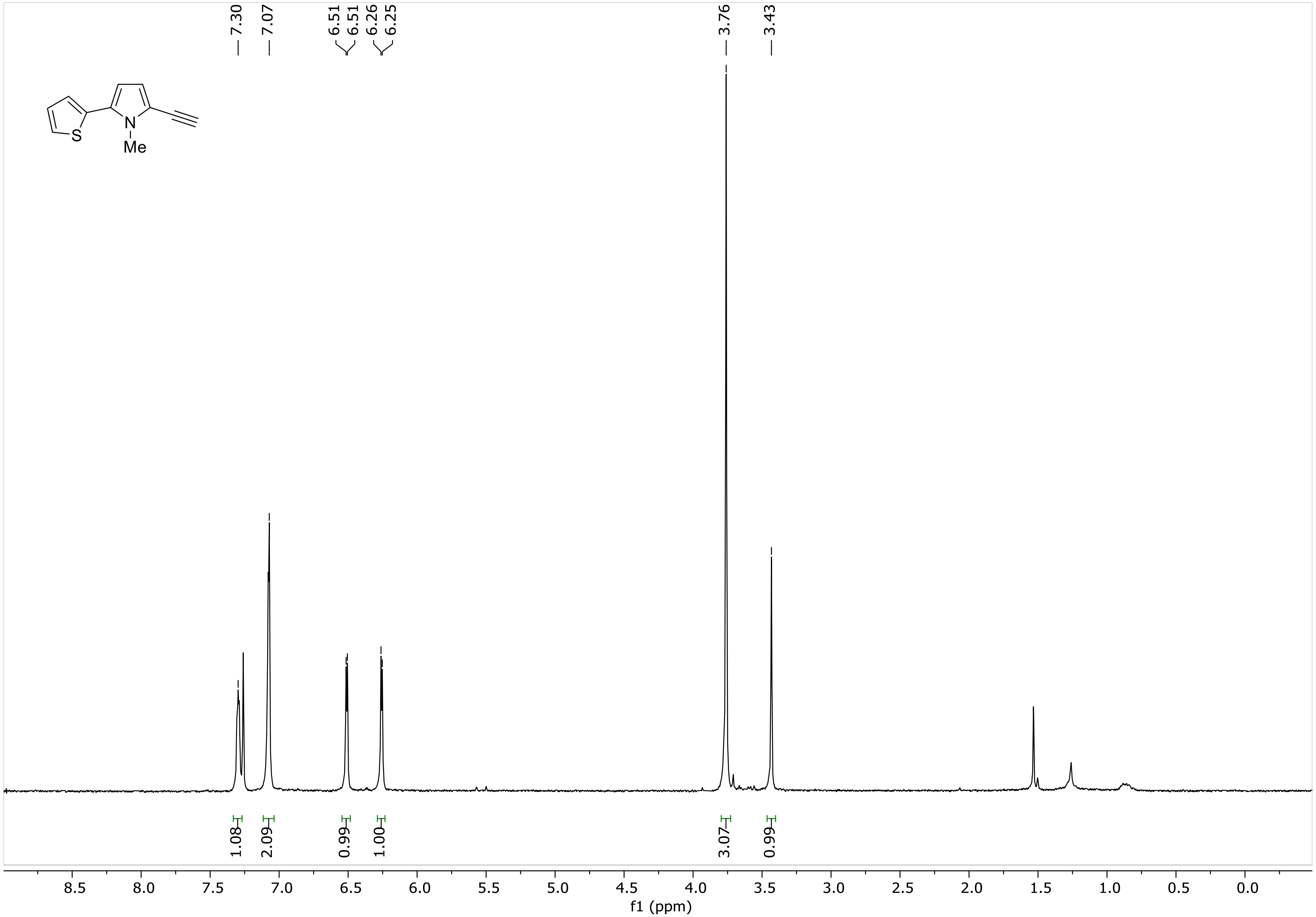

^{13}C NMR spectrum of 2-ethynyl-1-methyl-5-(thiophen-2-yl)-1*H*-pyrrole (**4j**) in $CDCl_3$

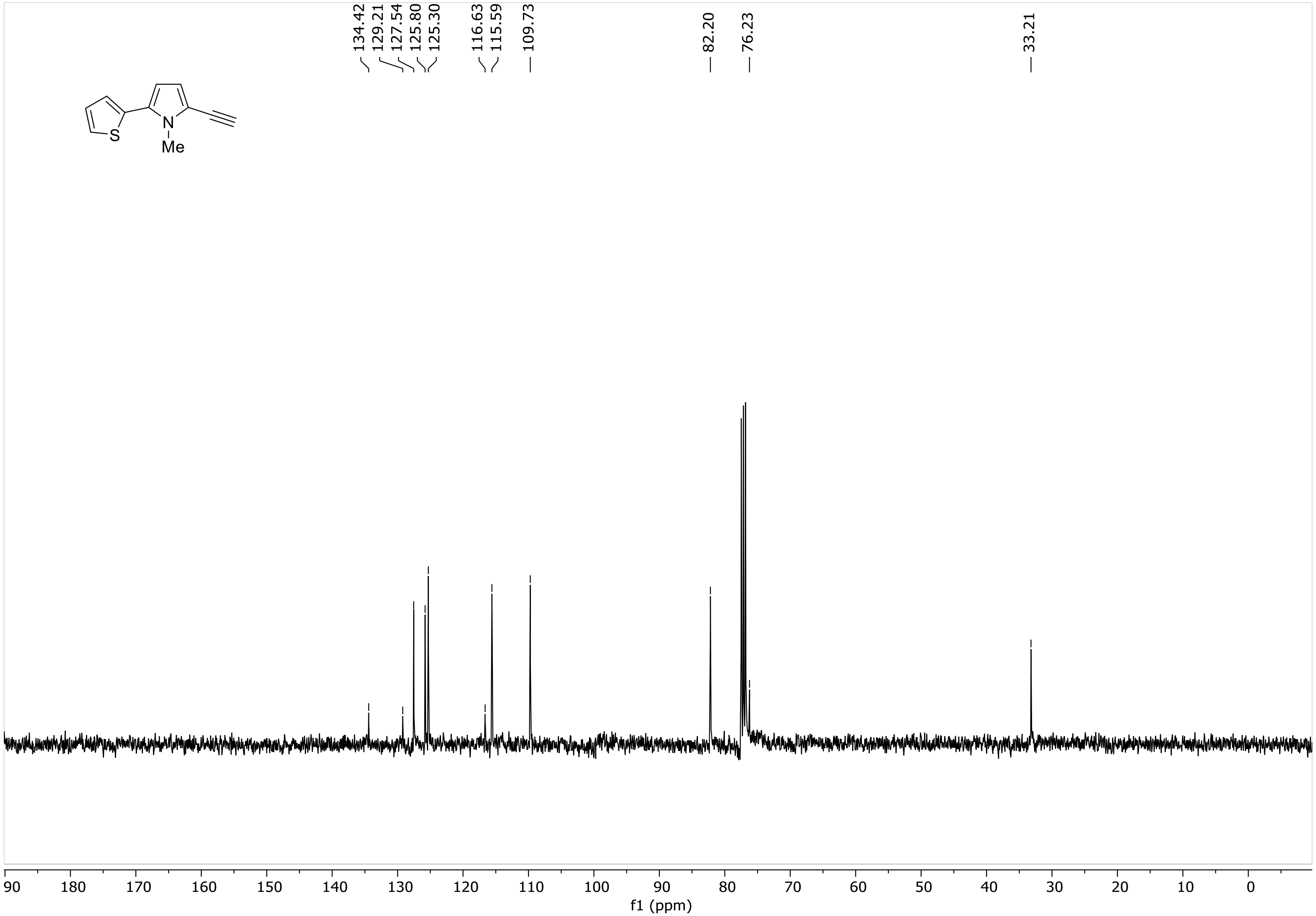

^{1}H NMR spectrum of 1-benzyl-2-ethynyl-1*H*-pyrrole (**4k**) in $CDCl_3$

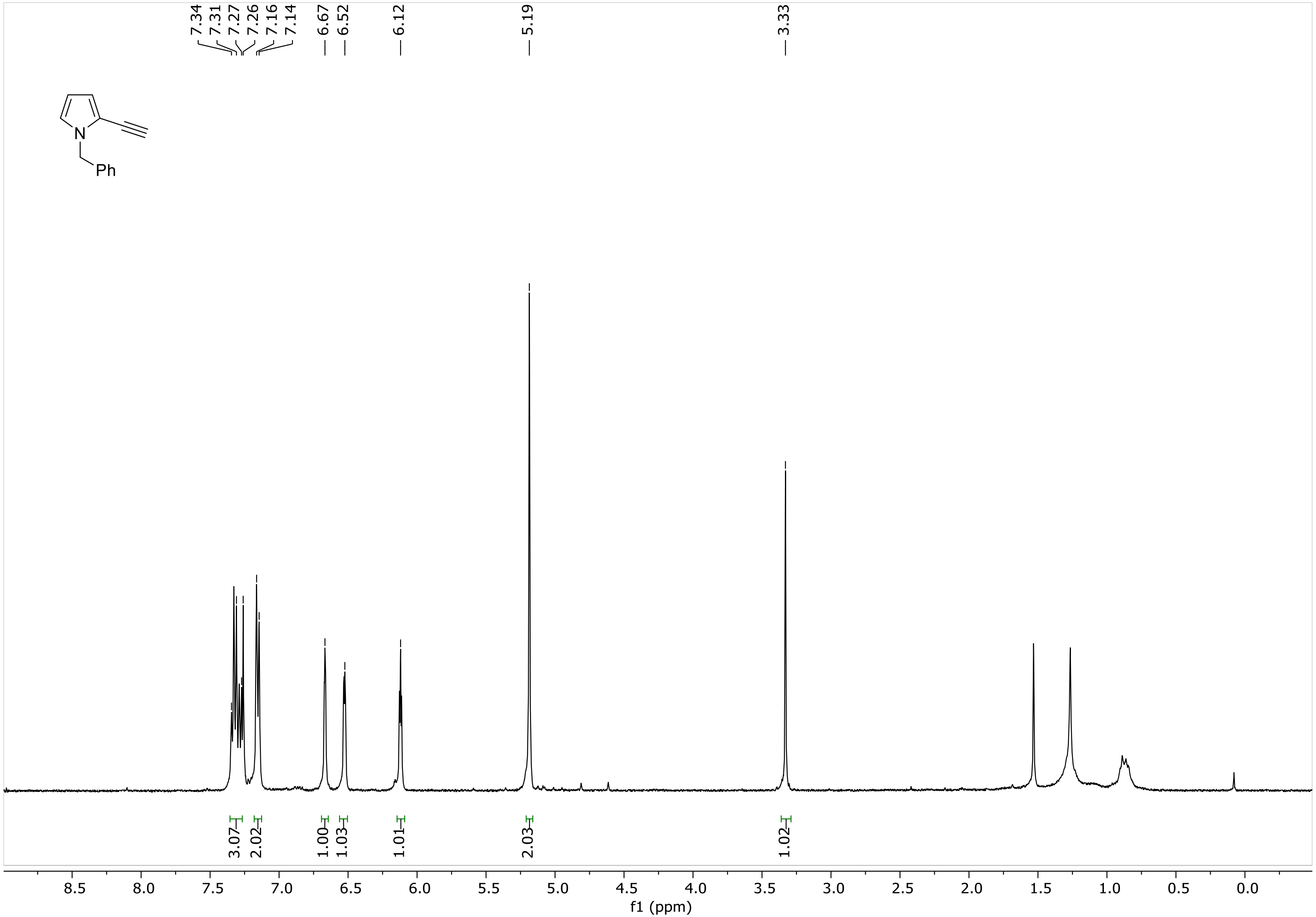

^{13}C NMR spectrum of 1-benzyl-2-ethynyl-1*H*-pyrrole (**4k**) in $CDCl_3$

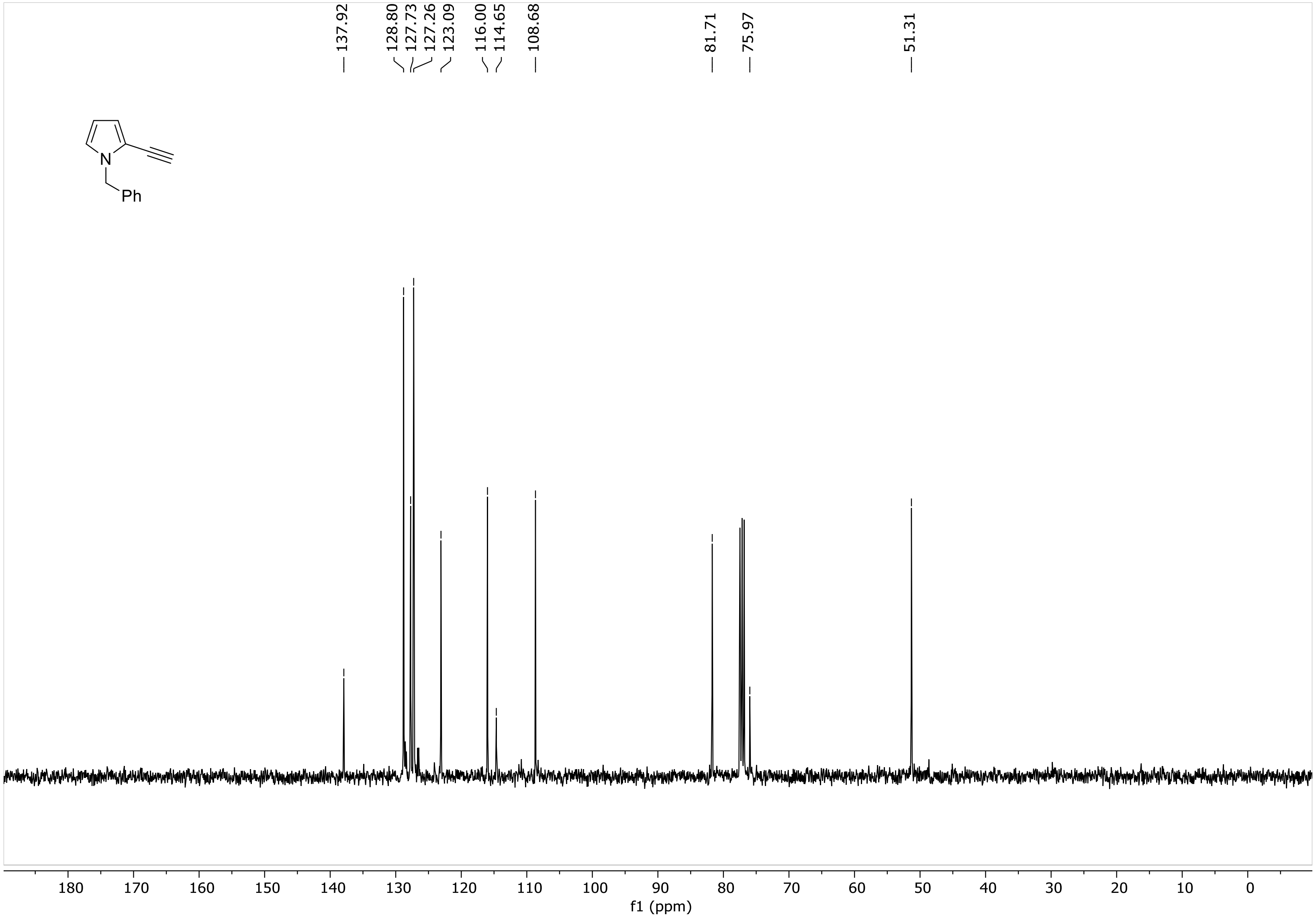

^{1}H NMR spectrum of 3-ethynyl-1-methyl-1*H*-indole (**6**) in $CDCl_3$

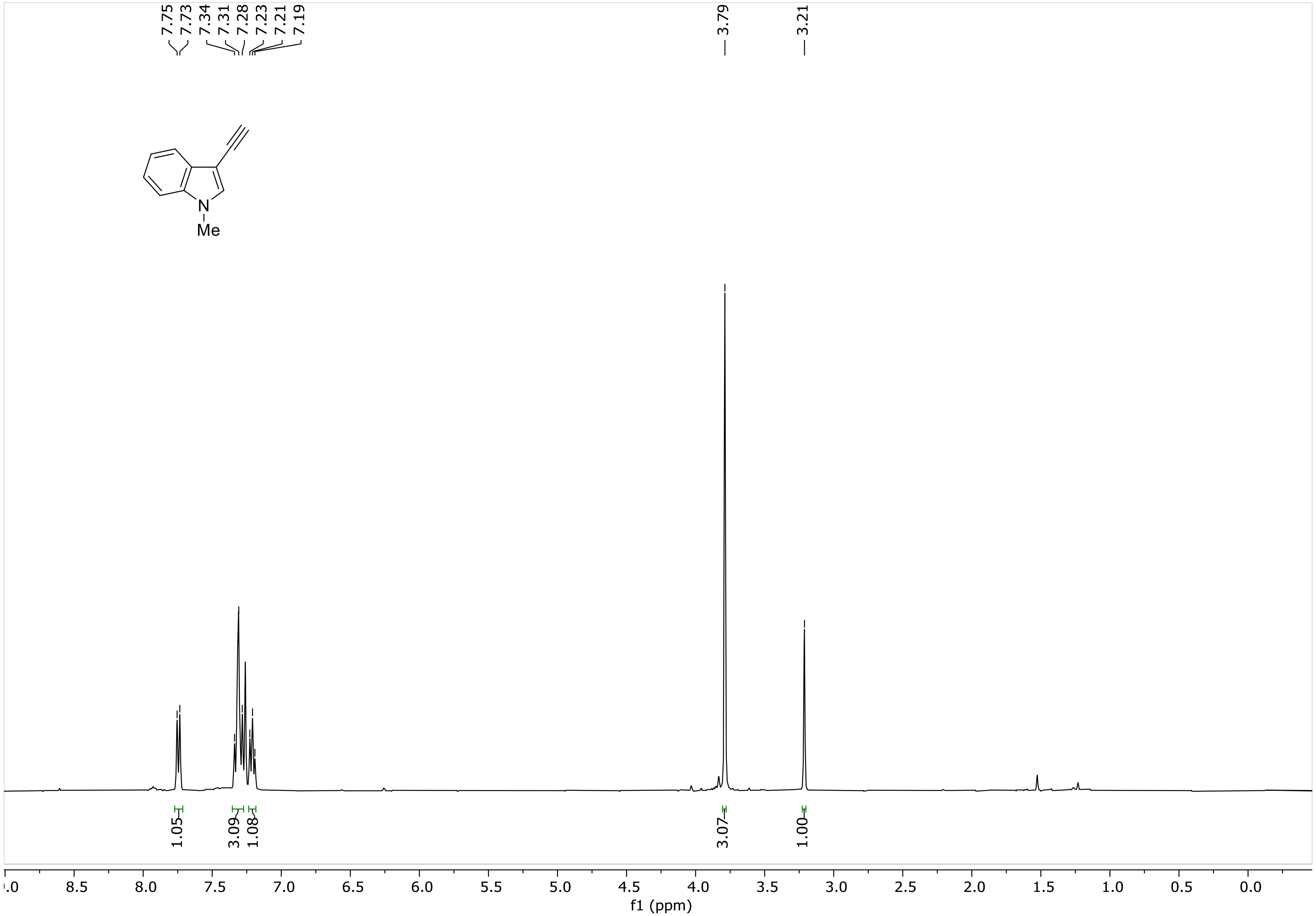

^{13}C NMR spectrum of 3-ethynyl-1-methyl-1*H*-indole (**6**) in $CDCl_3$

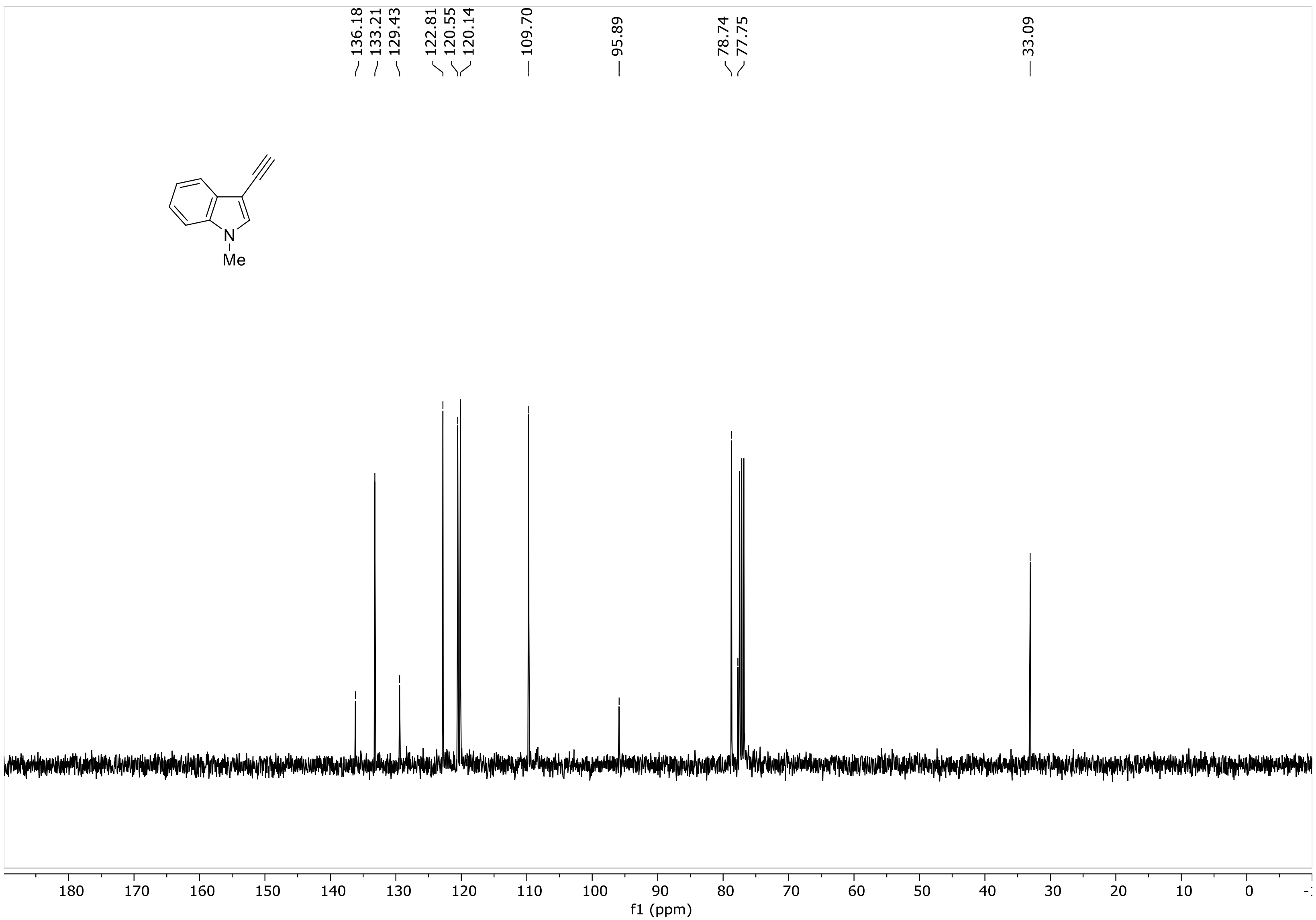

^{1}H NMR spectrum of 2-ethynyl-3,6-dimethyl-4,5,6,7-tetrahydrobenzofuran (**8**) in $CDCl_3$

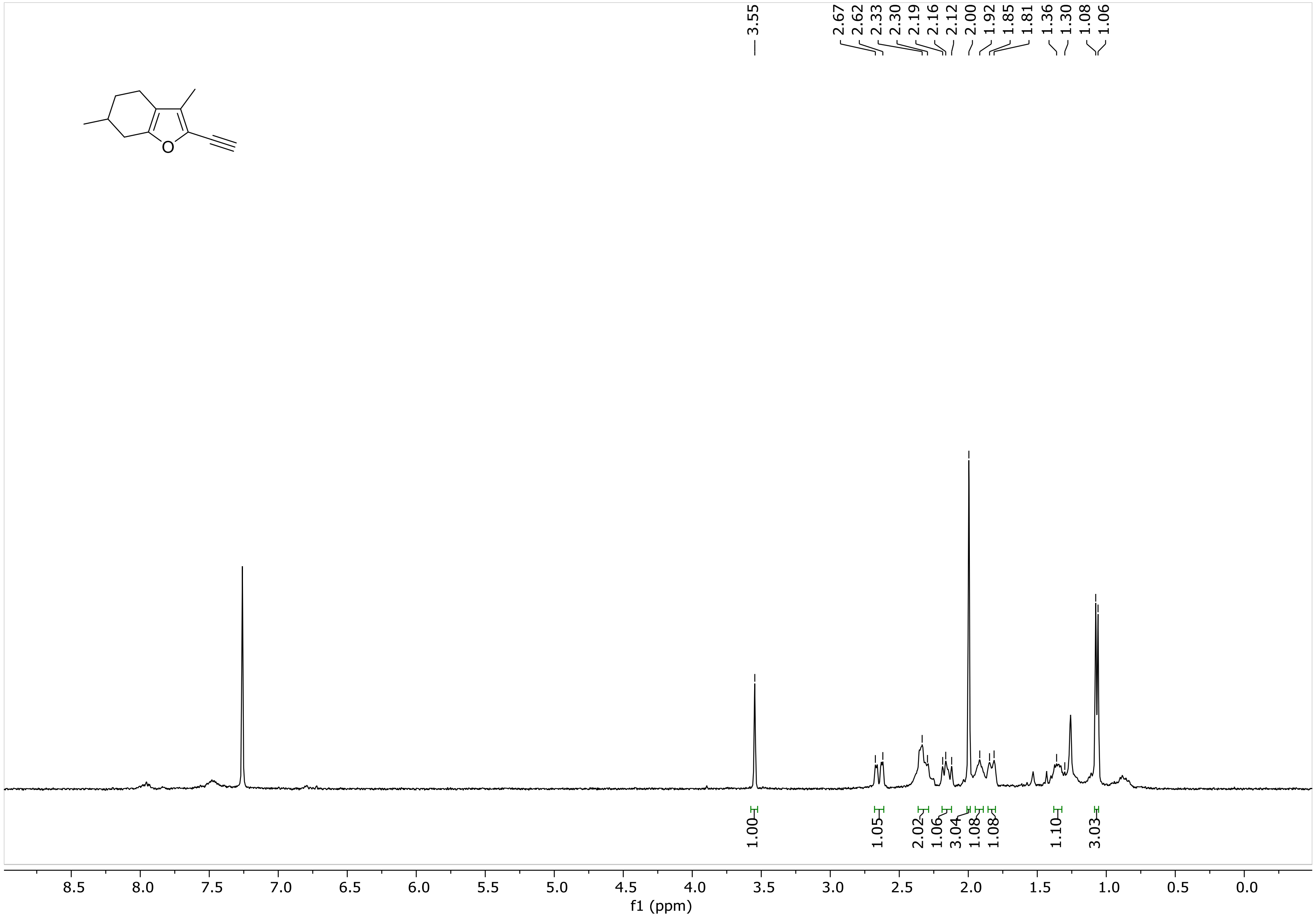

^{13}C NMR spectrum of 2-ethynyl-3,6-dimethyl-4,5,6,7-tetrahydrobenzofuran (**8**) in $CDCl_3$

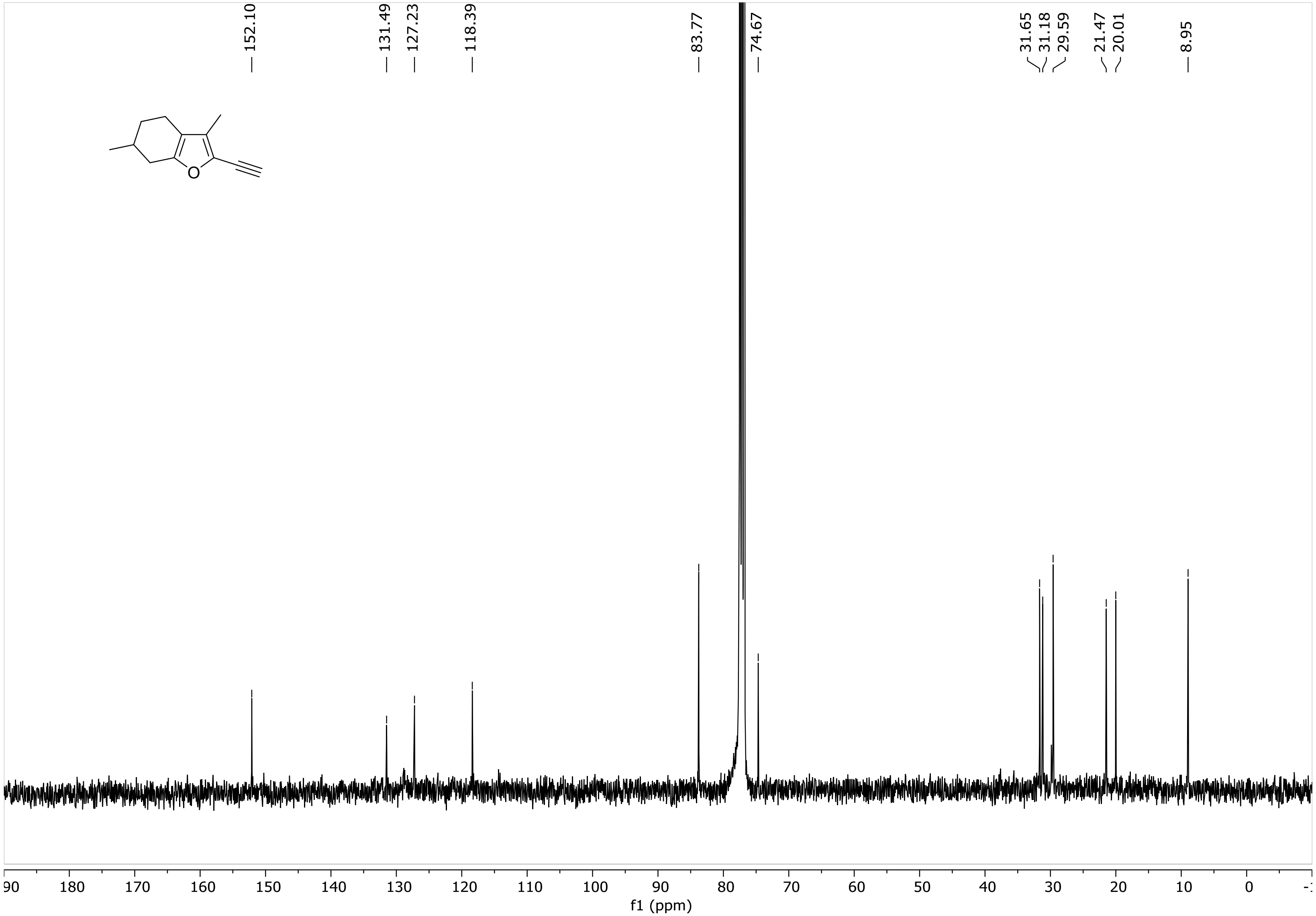

^{1}H NMR spectrum of 3-hydroxy-3-phenyl-5-(1-vinyl-4,5,6,7-tetrahydro-1*H*-indol-2-yl)pent-4-ynenitrile (**3c**) in $CDCl_3$

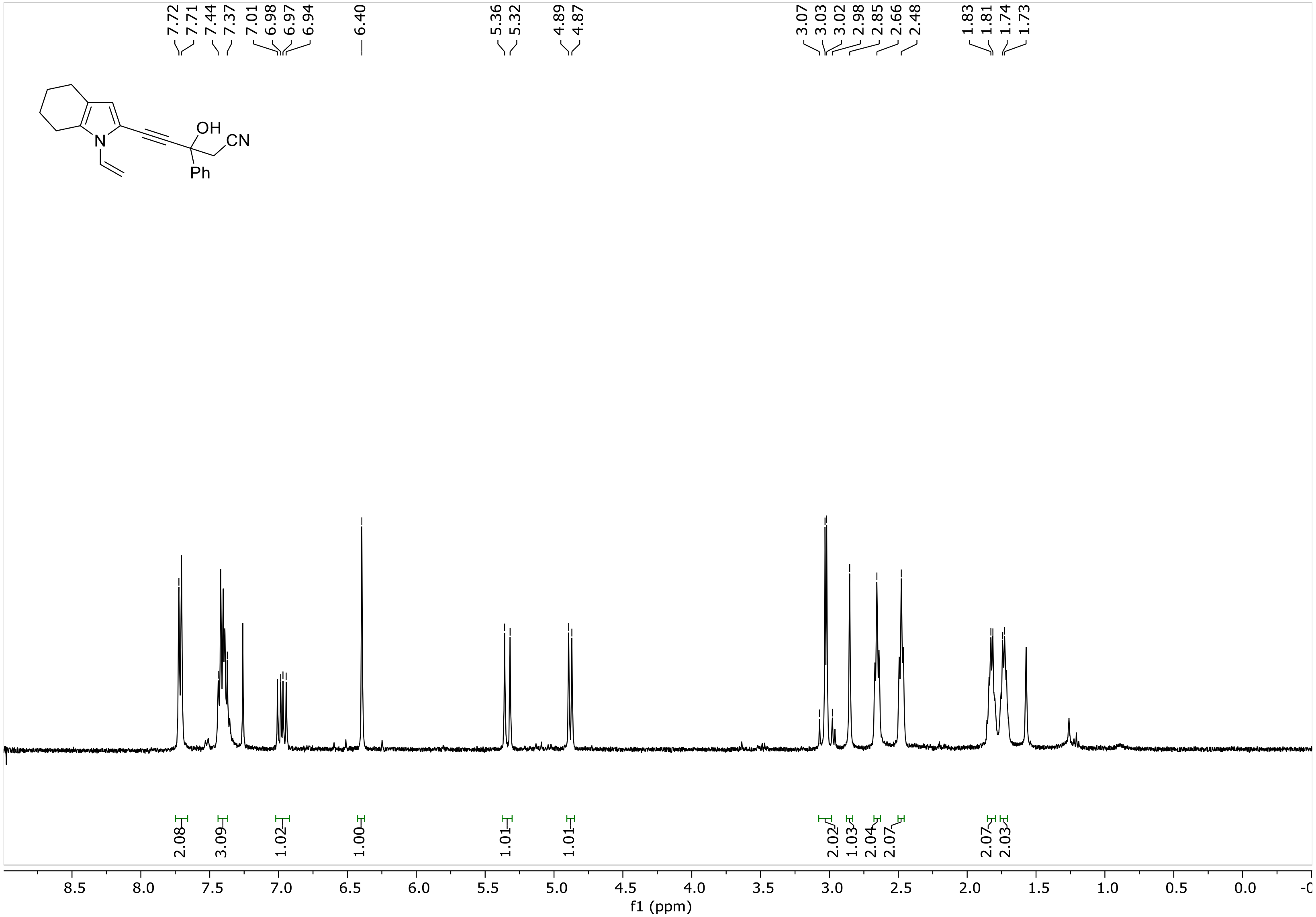

^{13}C NMR spectrum of 3-hydroxy-3-phenyl-5-(1-vinyl-4,5,6,7-tetrahydro-1*H*-indol-2-yl)pent-4-ynenitrile (**3c**) in $CDCl_3$

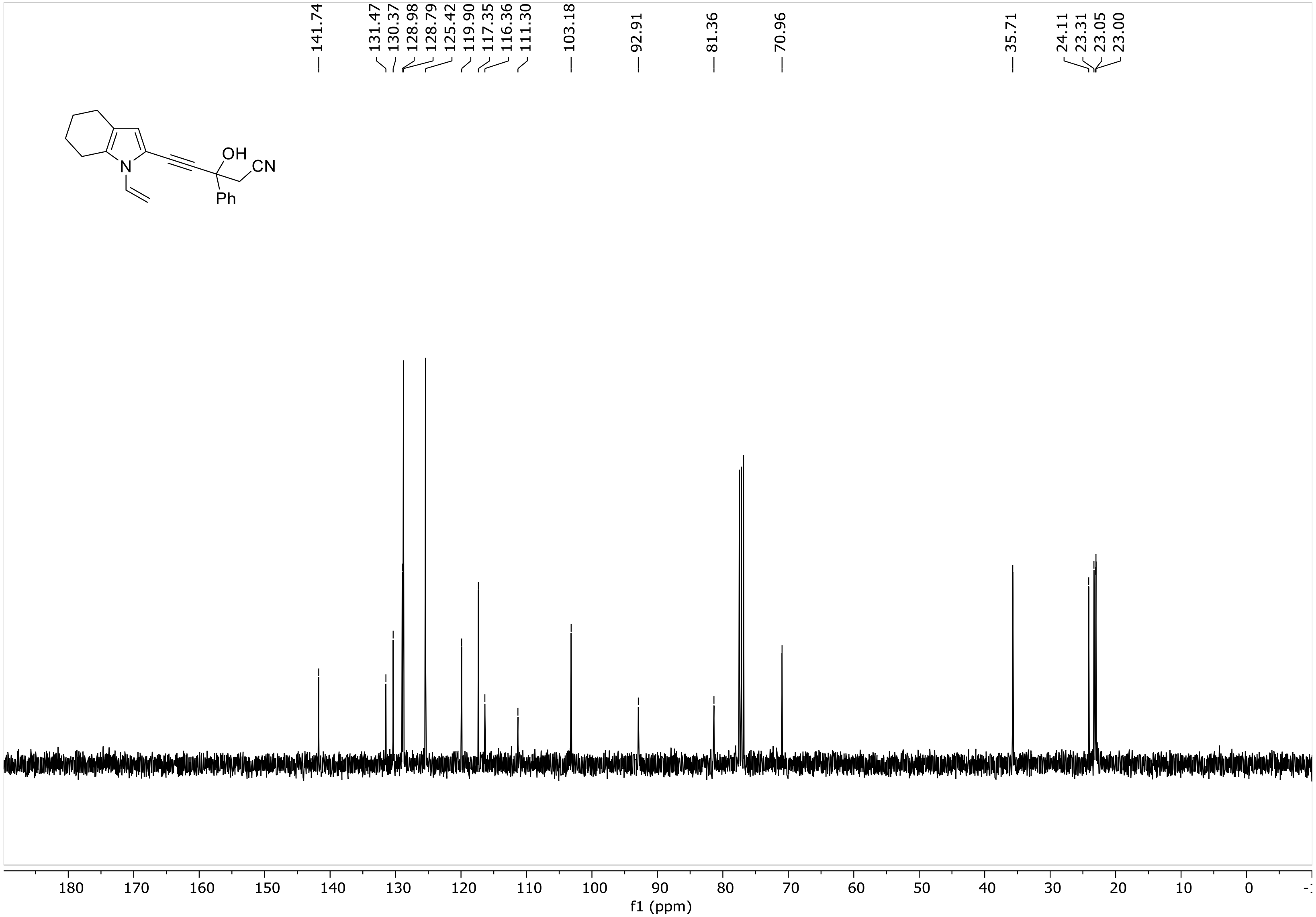

^{1}H NMR spectrum of 5-(4,5-dimethyl-1-vinyl-1*H*-pyrrol-2-yl)-3-hydroxy-3-phenylpent-4-ynenitrile (**3d**) in $CDCl_3$

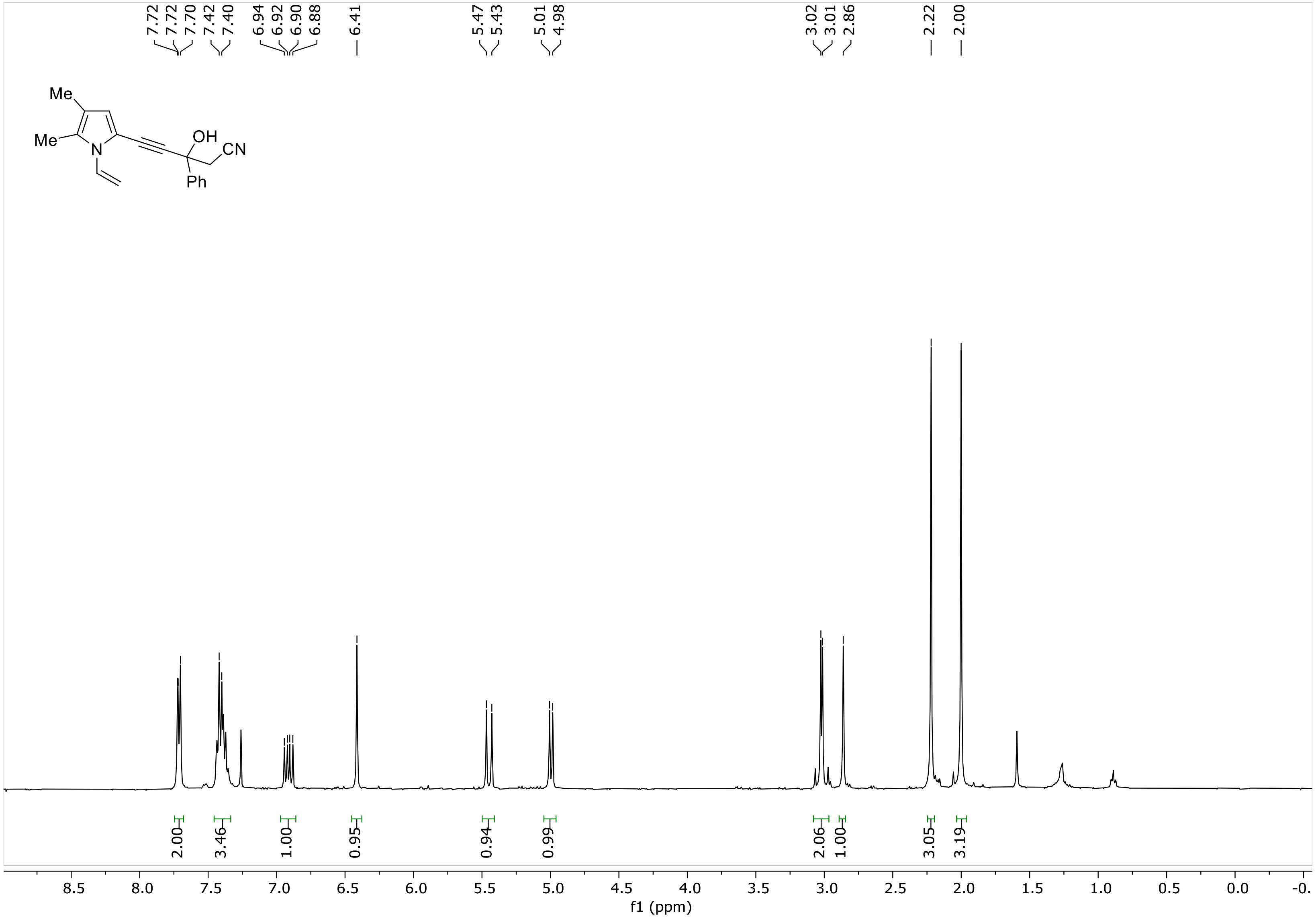

^{13}C NMR spectrum of 5-(4,5-dimethyl-1-vinyl-1*H*-pyrrol-2-yl)-3-hydroxy-3-phenylpent-4-ynenitrile (**3d**) in $CDCl_3$

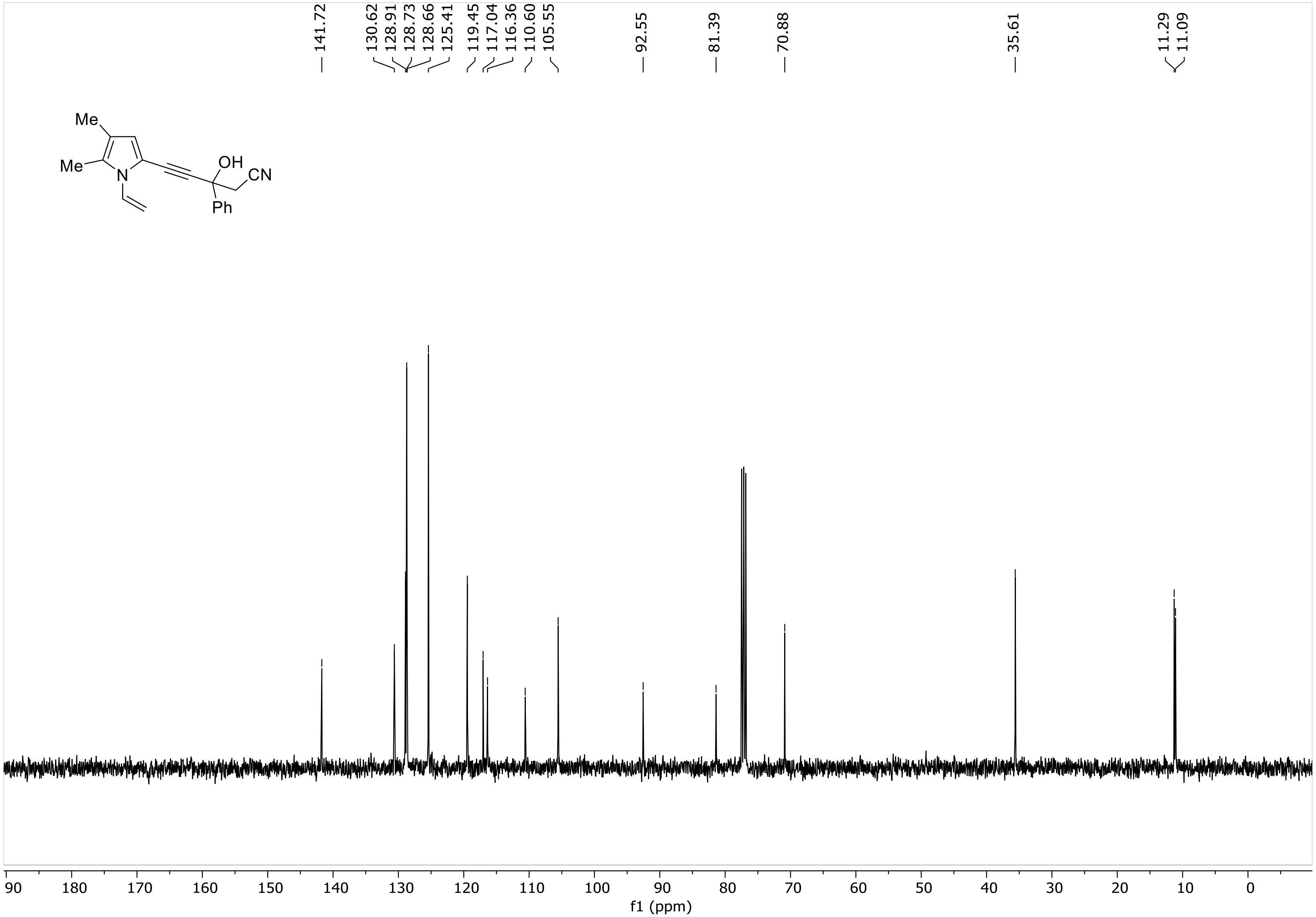

^{1}H NMR spectrum of 3-hydroxy-3-phenyl-5-(5-(p-tolyl)-1-vinyl-1*H*-pyrrol-2-yl)pent-4-ynenitrile (**3f**) in $CDCl_3$

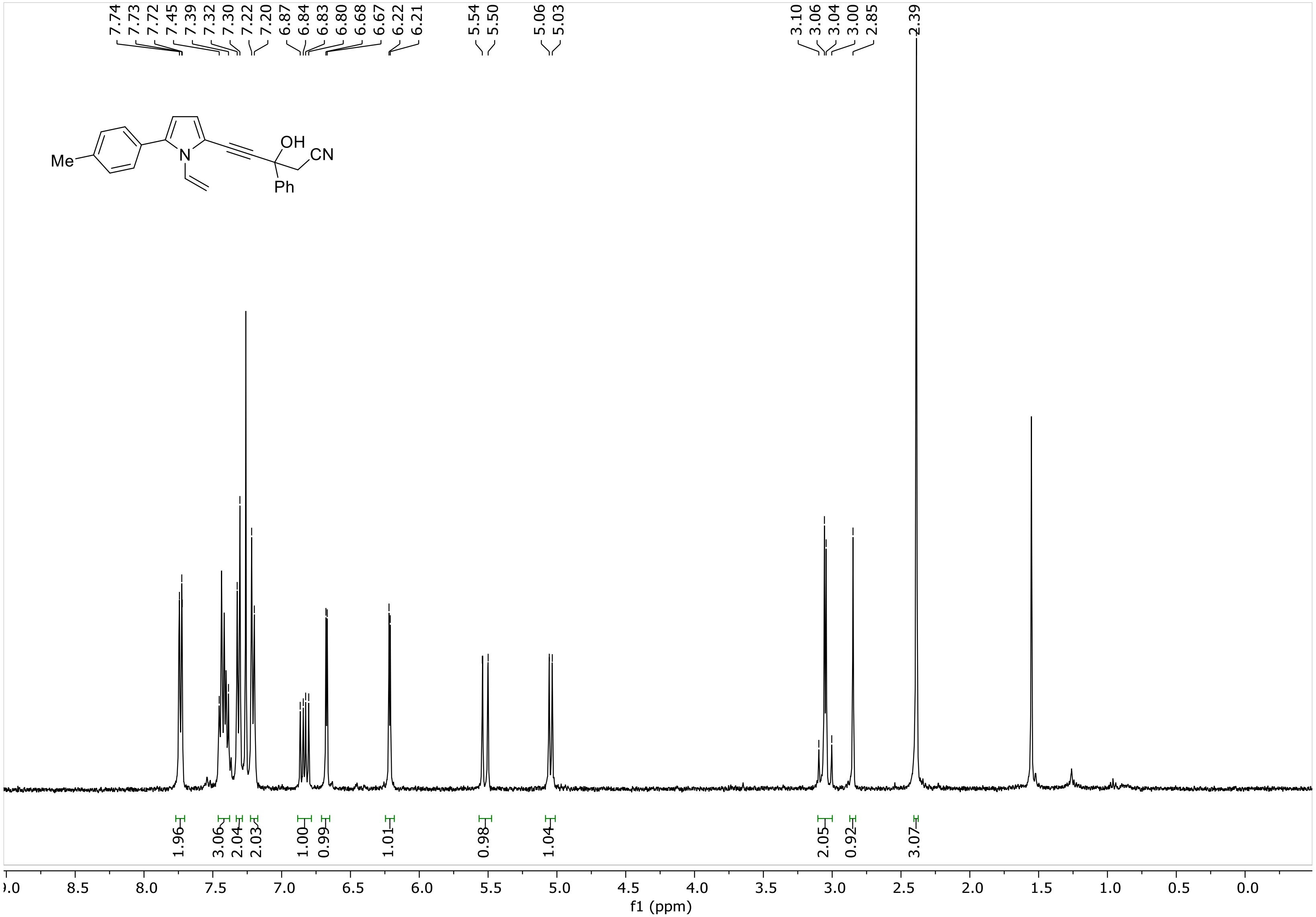

^{13}C NMR spectrum of 3-hydroxy-3-phenyl-5-(5-(p-tolyl)-1-vinyl-1*H*-pyrrol-2-yl)pent-4-ynenitrile (**3f**) in $CDCl_3$

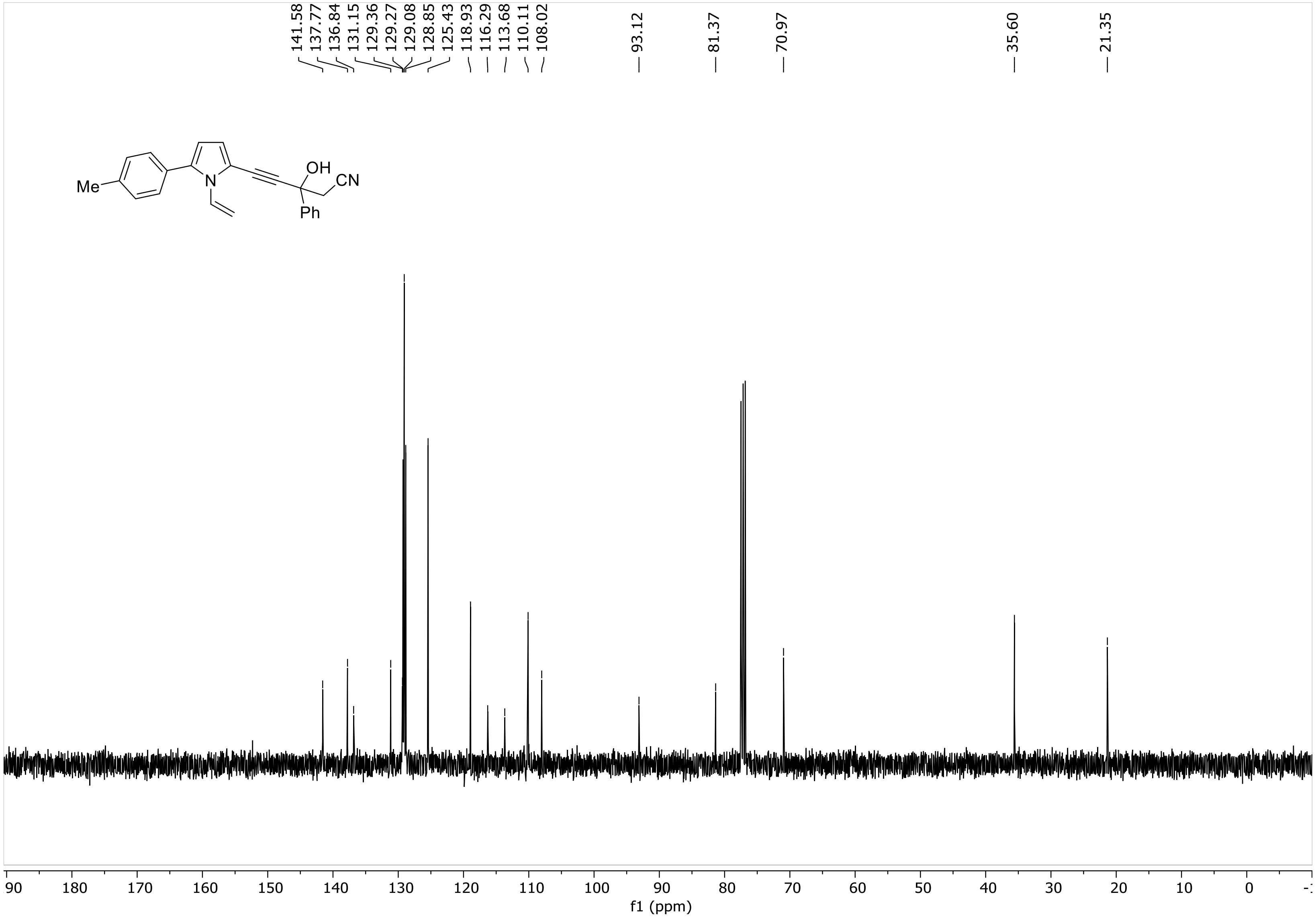

References

1. Gupton, J.T.; Telang, N.; Gazzo, D.F.; Barelli, P.J.; Lescalleet, K.E.; Fagan, J.W.; Mills, B.J.; Finzel, K.L.; Kanters, R.P.F.; Crocker, K.R.; et al. Preparation of indole containing building blocks for the regiospecific construction of indole appended pyrazoles and pyrroles. *Tetrahedron* **2013**, *69*, 5829–5840. https://doi.org/10.1016/j.tet.2013.05.045.
2. Tomilin, D.N.; Sobenina, L.N.; Saliy, I.V.; Ushakov, I.A.; Belogolova, A.M.; Trofimov, B. A. Substituted pyrrolyl-cyanopyridines on the platform of acylethynylpyrroles via their 1:2 annulation with acetonitrile under the action of lithium metal. *New J. Chem.* **2022**, *46*, 13149–13155. https://doi.org/10.1039/D2NJ02011D.
3. Grimme, S. Semiempirical hybrid density functional with perturbative second-order correlation. *J. Chem. Phys.* **2006**, *124*, 034108. https://doi.org/10.1063/1.2148954.
4. Becke, A.D. Density-functional thermochemistry. III. The role of exact exchange. *J. Chem. Phys.* **1993**, *98*, 5648–5652. https://doi.org/10.1063/1.464913.
5. Lee, C.; Yang, W.; Parr, R.G. Development of the Colle-Salvetti correlation-energy formula into a functional of the electron density. *Physical Review B* **1988**, *37*, 785–789. https://doi.org/10.1103/PhysRevB.37.785.
6. Krishnan, R.; Binkley, J.S.; Seeger, R.; Pople, J.A. Self-consistent molecular orbital methods. XX. A basis set for correlated wave functions. *J. Chem. Phys.* **1980**, *72*, 650–654. https://doi.org/10.1063/1.438955.
7. Tomasi, J.; Mennucci, B.; Cammi, R. Quantum Mechanical Continuum Solvation Models. *Chem. Rev.* **2005**, *105*, 2999–3094. https://doi.org/10.1021/cr9904009.
8. Cossi, M.; Rega, N.; Scalmani, G.; Barone, V. Energies, structures, and electronic properties of molecules in solution with the C-PCM solvation model. *Journal of Computational Chemistry* **2003**, *24*, 669–681. https://doi.org/10.1002/jcc.10189.
9. Vitkovskaya, N.M.; Orel, V.B.; Absalyamov, D.Z.; Trofimov, B.A. Self-Assembly of *N*-Phenyl-2,5-dimethylpyrrole from Acetylene and Aniline in KOH/DMSO and $KOBu^t$/DMSO Superbase Systems: A Quantum-Chemical Insight. *J. Org. Chem.* **2020**, *85*, 10617–10627. https://doi.org/10.1021/acs.joc.0c01185.
10. Frisch, M.J.; Trucks, G.W.; Schlegel, H.B.; Scuseria, G.E.; Robb, M.A.; Cheeseman, J.R.; Scalmani, G.; Barone, V.; Mennucci, B.; Petersson, G.A.; et al. *Gaussian 09*, Gaussian, Inc.: Wallingford, CT, USA, 2009.
11. Petersson, G.A. Complete Basis Set Models for Chemical Reactivity: from the Helium Atom to Enzyme Kinetics. In *Quantum-Mechanical Prediction of Thermochemical Data*, Cioslowski, J., Ed. Springer Netherlands: Dordrecht, 2001; pp 99–130.
12. Montgomery Jr., J.A.; Frisch , M.J.; Ochterski, J.W.; Petersson, G.A. A complete basis set model chemistry. VII. Use of the minimum population localization method. *J. Chem. Phys.* **2000**, *112*, 6532–6542. https://doi.org/10.1063/1.481224.
